# The RNA-dependent association of phosphatidylinositol 4,5-bisphosphate with intrinsically disordered proteins contribute to nuclear compartmentalization

**DOI:** 10.1371/journal.pgen.1011462

**Published:** 2024-12-02

**Authors:** Martin Sztacho, Jakub Červenka, Barbora Šalovská, Ludovica Antiga, Peter Hoboth, Pavel Hozák

**Affiliations:** 1 Department of Biology of the Cell Nucleus, Institute of Molecular Genetics of the Czech Academy of Sciences, Prague, Czech Republic; 2 Laboratory of Cancer Cell Architecture, Institute of Biochemistry and Experimental Oncology, First Faculty of Medicine, Charles University, Prague, Czech Republic; 3 Laboratory of Applied Proteome Analyses, Research Center PIGMOD, Institute of Animal Physiology and Genetics of the Czech Academy of Sciences, Liběchov, Czech Republic; 4 Laboratory of Proteomics, Institute of Biochemistry and Experimental Oncology, First Faculty of Medicine, Charles University, Prague, Czech Republic; 5 Department of Genome Integrity, Institute of Molecular Genetics of the Czech Academy of Sciences, Prague, Czech Republic; 6 Yale Cancer Biology Institute, Yale University School of Medicine, West Haven, Connecticut, United States of America; Friedrich-Schiller-Universitat Jena, GERMANY

## Abstract

The RNA content is crucial for the formation of nuclear compartments, such as nuclear speckles and nucleoli. Phosphatidylinositol 4,5-bisphosphate (PIP2) is found in nuclear speckles, nucleoli, and nuclear lipid islets and is involved in RNA polymerase I/II transcription. Intriguingly, the nuclear localization of PIP2 was also shown to be RNA-dependent. We therefore investigated whether PIP2 and RNA cooperate in the establishment of nuclear architecture. In this study, we unveiled the RNA-dependent PIP2-associated (RDPA) nuclear proteome in human cells by mass spectrometry. We found that intrinsically disordered regions (IDRs) with polybasic PIP2-binding K/R motifs are prevalent features of RDPA proteins. Moreover, these IDRs of RDPA proteins exhibit enrichment for phosphorylation, acetylation, and ubiquitination sites. Our results show for the first time that the RDPA protein Bromodomain-containing protein 4 (BRD4) associates with PIP2 in the RNA-dependent manner via electrostatic interactions, and that altered PIP2 levels affect the number of nuclear foci of BRD4 protein. Thus, we propose that PIP2 spatiotemporally orchestrates nuclear processes through association with RNA and RDPA proteins and affects their ability to form foci presumably via phase separation. This suggests the pivotal role of PIP2 in the establishment of a functional nuclear architecture competent for gene expression.

## Author summary

Our research focuses on how different molecules in the nucleus of human cells work together to organize nuclear compartments. We focused on phosphatidylinositol 4,5-bisphosphate (PIP2), a lipid present in various nuclear structures, and its relationship with RNA and associated proteins. Previous studies suggested that the presence of PIP2 in the nucleus was dependent on RNA, which led us to investigate it further. We used advanced techniques to identify proteins associated with PIP2 and RNA in human cells. Our results show that these proteins often have regions known to specifically bind PIP2. A key protein in our study, BRD4, which is critical for gene expression, was found to bind PIP2 in an RNA-dependent manner. Variations in PIP2 levels significantly affect the number of BRD4 foci in the nucleus. Our study suggests that PIP2 is essential for organizing the nuclear environment by interacting with RNA and specific proteins to influence gene expression. This finding helps us understand the complex organization within the nucleus that is essential for proper cellular function.

## Introduction

Differentially phosphorylated inositol headgroups of different phosphoinositides (PIPs) serve as a recognition code for the recruitment of a plethora of interacting proteins [1–4]. PIPs are typically embedded in eukaryotic cell membranes where they regulate processes such as vesicular trafficking, actin polymerization, or autophagy [5,6]. The pioneering work on identifying the presence of PIPs in the cell nucleus was done several decades ago [7–10] but is now receiving increasing attention. One of the most widely studied nuclear PIPs is phosphatidyl inositol 4,5-bisphosphate (PIP2). PIP2 localizes to nuclear speckles, nucleoli, and small nucleoplasmic structures called nuclear lipid islets (NLIs) [11,12]. Nuclear PIPs are involved in gene expression [2,11,13]. In particular, nuclear PIP2 regulates transcription by affecting the condensation capacity of the RNA Pol2 initiation complex [14]. Interestingly, nuclear compartments containing PIP2, such as the aforementioned nuclear speckles, nucleoli, and nucleoplasmic transcription initiation foci, are formed by the process of phase separation [15–21].

The phase separation-driven formation of membraneless compartments, sometimes referred to as ’biomolecular condensates’, is associated with enhanced kinetics of biochemical reactions in the living cell [22–25]. The formation of these compartments provides high local concentrations of reaction components and forms diffusion barriers that serve as adsorption catalyst surfaces [26]. In addition, biomolecular condensates allow the sequential progression of processes through the successive coupling of subsequent reactions in multilayered compartmentalized reaction chambers, such as ribosomal biogenesis in nucleoli, packaging of hnRNP particles in Cajal bodies, or the involvement of nuclear speckles in pre-mRNA splicing [17,27–29].

The formation of phase-separated biomolecular condensates can be mathematically described and computationally modeled using the theory of stickers and linkers [30]. Stickers are local modules that allow multivalent intra- and intermolecular interactions and are represented by classical globular domains of proteins or by stretches of charged amino acids connected by flexible linker regions in intrinsically disordered regions (IDRs) [31,32]. Condensation of IDR-containing proteins is often driven by charged amino acid stretches within IDRs [33–35]. In addition, it has been previously described that changes in net charge and amino acid types within an IDR can even navigate proteins to different core regions [33]. Thus, both the amino acid composition and the posttranslational modifications (PTMs), which together generate the charge pattern of the IDR, are important determinants of sub-nuclear protein localization [35]. Electrostatic interactions appear to be fundamental determinants of condensate properties [36,37]. Thus, negatively charged polymeric molecules such as RNA are important factors in the formation and dissolution of some nuclear condensates.

Indeed, the biomolecular condensates in the nucleus are typically formed by low-affinity multivalent interactions between proteins and RNA [38,39]. RNA has a positive or negative effect on condensate formation, depending on the situation and the type of RNA [40–42]. The short RNA molecules buffer and thus reduce the local tendency to form a condensate. Conversely, longer RNA molecules often increase condensate formation [40,41,43,44]. In addition, PTMs such as phosphorylation are another important regulatory step affecting condensate formation or dissolution [18,33,40,45]. The interaction between higher-order folded RNA and lipid molecules has been suggested previously [21,46–52]. Higher-order RNA has a scaffolding function that brings together RNA-binding proteins to form nuclear subcompartments [53–57]. The formation of these RNA folds depends on intra- and intermolecular double-stranded RNA (dsRNA) duplexes. However, a general mechanism or identification of common mechanistic principles has been lacking.

We hypothesized that negatively charged nuclear PIPs are interesting candidates for the regulation of biomolecular condensation via phase separation. PIPs provide a platform for the recruitment of interacting proteins, thereby increasing their local concentration. RNA and PIPs may cooperate in the formation of condensates, such as in the process of influenza virus particle biogenesis [58]. PIPs carry a negative charge, which could ultimately alter the overall net charge of condensates and thus influence condensate formation and size. Therefore, the possible spatial interplay between nuclear PIPs and RNA in regulating condensation seems plausible. Indeed, a recent study showed that not only proteins and RNA but also metabolites including phospholipids (e.g., PIPs) are enriched in condensates [59]. We have previously shown that RNA is important for nuclear PIP2 levels, as RNA removal by RNase A dramatically decreased the PIP2 signal measured by immunofluorescence [11]. In the current study, we speculate that the higher-order RNA might be responsible for the correct localization of PIP2 in the eukaryotic nucleus. Therefore, we used bacterial RNase III, normally associated with siRNA processing, to remove short dsRNA regions followed by quantitative mass spectrometry (MS) proteomic analysis of the nuclear fraction. We identified the RNA-dependent PIP2-associated (RDPA) nuclear proteome and performed bioinformatic analyses of the physicochemical properties of the identified proteins. Subsequently, we proposed and successfully validated a model in which nuclear PIP2 may serve as a recognition motif that regulates the formation of Bromodomain-containing protein 4 (BRD4 protein) foci. These are known to form by phase separation and to compartmentalize and concentrate the transcriptional apparatus. Thus, PIP2 may play an important role in the establishment of nuclear architecture.

Our results suggest a molecular mechanism in which PIP2 acts as a molecular wedge for the recruitment of the lysin/arginine (K/R) motif-containing RDPA protein BRD4 by higher-order RNA molecules. This presumably leads to local regulation of the condensation potential, as manifested by different numbers of BRD4 foci when PIP2 levels are high. Nuclear PIP2 levels may therefore dictate where certain RDPA proteins accumulate and potentially form condensate. Thus, changes in the localization and condensation potential of RDPA proteins could affect the rates of the biochemical reactions involved. Taken together, our data demonstrated the formation of a specific type of biomolecular condensates via the association of complexes formed by RNA, proteins, and PIP2, and are therefore relevant to our understanding of the principles underlying the establishment of functional nuclear compartments.

## Material and methods

### Cell culture, antibodies

HeLa (ATCC no. CCL2) cells were cultured in suspension in DMEM media (Sigma D6429) supplemented with 10% fetal bovine serum in spinner flasks at 37°C 10% CO_2_ atmosphere for 5 days. U2OS (ATCC no. HTB96) were grown in DMEM media (Sigma D6429) with 10% FBS at 37°C in a humidified 5% CO_2_ atmosphere. Antibodies were used in this study at concentrations according to the manufacturer’s instructions (Table 1).

**Table 1:** Antibodies used in this study.

Antibody	Vendor	Ref number	Immunofluorescence	Western Blot
Anti-PIP2	Echelon Biosciences Inc., USA	AB010220-28	5 μg/mL	
Anti-BRD4	Sigma-Aldrich, St. Louis, MO, USA	HPA015055	0.20 μg/mL	0.20 μg/mL
Anti-SON	Sigma-Aldrich, St. Louis, MO, USA	HPA062997	1 μg/mL	
Anti-PIP5KA	Sigma-Aldrich, St. Louis, MO, USA	HPA029366		0.10 μg/mL
Anti-SHIP2	Abcam, UK	Ab70267		0.50 μg/mL
Anti-actin	Sigma-Aldrich, St. Louis, MO, USA	MABT219		1.7 μg/mL
Anti-GST	Abcam, UK	Ab6613		2 μg/mL
Anti-CAND1	Sigma-Aldrich, St. Louis, MO, USA	HPA-069053		0.2 μg/mL
IRDye 800 CW Donkey anti-Rabbit IgG	LI-COR Biosciences, Lincoln, NE, USA	926–32213		0.10 μg/mL
IRDye 800 CW Donkey anti-Mouse IgG	LI-COR Biosciences, Lincoln, NE, USA	926–32212		0.10 μg/mL
IRDye 680RD Donkey anti-Mouse IgG	LI-COR Biosciences, Lincoln, NE, USA	926–68072		0.10 μg/mL
IRDye 680RD Donkey anti-Rabbit IgG	LI-COR Biosciences, NE, USA	926–68073		0.10 μg/mL
Goat anti-Mouse IgG (H+L) Highly Cross-Adsorbed, Alexa Fluor 568	Invitrogen, MA, USA	A-11031	5 μg/mL	
Goat anti-Rabbit IgG (H+L) Highly Cross-Adsorbed, Alexa Fluor 488	Invitrogen, MA, USA	A-11034	5 μg/mL	

### Immunofluorescence labelling

U2OS cells were grown on high-performance cover glasses of 12 mm in diameter with restricted thickness-related tolerance (depth = 0.17 mm ± 0.005 mm) and the refractive index = 1.5255 ± 0.0015 (Marienfeld 0107222). The cells were fixed with 4% formaldehyde for 20 min, permeabilized with 0.1% Triton X-100 for 5 min and three times washed in phosphate-buffered saline (PBS). Specimens were blocked by 5% bovine serum albumin (BSA) in PBS for 30 min. The specimens were incubated 1 h with primary antibodies (Table 1) or GST-PLCδ1 PH domain (1 μg/μl) followed by GST specific primary antibody, three times washed with PBS and subsequently incubated 30 min with respective secondary antibodies (Table 1). Followed by three PBS washes and one wash with tap water, the specimens were air-dried for 20 min and mounted in 90% glycerol with 4% n-propyl gallate (NPG) media.

### Nuclear RNA extraction

Suspension HeLa cells were grown in 500 mL spinner flasks in DMEM media and harvested by 800 g centrifugation for 15 min at 4°C. HeLa cell pellets were resuspended in 3 mL of lysis buffer (50 mM Hepes pH 7.4, 150 mM NaCl, 2 mM MgCl_2_, 0.5% NP-40) with 20 U RNase inhibitor (Applied Biosystem, MA, USA, S17857). Sample was spun down at 1000 g, 4°C for 5 min. Supernatant representing cytoplasmic fraction was taken out. Additional 3 mL of lysis buffer were added and spun at 1000 g, 4°C for 1 min and supernatant was discarded. Additional 1.2 mL of lysis buffer was added to pellet and mixed. After that step, 2.5 mL of TRIzol (Sigma, MO, USA, BCCF2003) and 2.5 mL of chloroform were added into the sample and vortexed vigorously. Sample was centrifuged at 12,000 g for 5 min at 4°C. The upper phase was taken out and transferred to a new tube, 0.7 volume of isopropanol was added and mixed. The sample was centrifuged at 12,000 g for 15 min at 4°C. Supernatant was removed, the pellet was washed by 80% ethanol and aspirated. The pellet was air dried for 10 min, dissolved in water and RNA concentration was measured by NanoDrop. The DNA was cleaved out by incubation with DNase I enzyme, when 160 μg of RNA was mixed with 12 μL of supplied reaction buffer and 30 U of DNase I (Sigma, MO, USA, D4527). The reaction was incubated for 30 min at 37°C, then mixed with 0.8 volume of isopropanol and centrifuged at 12,000 g for 15 min at 4°C. Pellet was washed with 80% of ethanol, air-dried for 10 min at RT, and dissolved in RNA-free water. The concentration of RNA was determined by NanoDrop measurement.

### RNase III treatment of U2OS cells

To obtain semi-permeabilized cells, 90% confluent U2OS cell cultures were washed twice with PBS. Cells were permeabilized by 0.1% Triton X-100 at RT in buffer (20 mM Hepes pH 7.4, 150 mM NaCl, 25% glycerol, 1 mM DTT, cOmplete EDTA-free protease inhibitor cocktail (La Roche Ltd., Basel, Switzerland, 05056489001). Semi-permeabilized and non-permeabilized cells were treated for 10 min at RT by RNase III enzyme (2 U of RNase III (Short Cut, New England BioLabs, Massachusetts, USA, M0245S) with 20 mM MnCl2 in 1× reaction ShortCut buffer). This step was followed by five times wash in PBS and fixation by 4% PFA for 15 min at RT. Cells were than permeabilized by 0.2% Triton X-100 for additional 15 min at RT. After three PBS washes, the cells were blocked for 30 min in 3% BSA in PBS and subjected to immunofluorescence protocol. Measured integral signal density of nuclear PIP2 signal was analyzed by FIJI software [60]. Data were obtained from three biological replicates, in total 76 cells were quantified for non-treated control cells condition, and 89 cells were quantified for RNase III treated cells condition and N = 58 RNase III-treated non-permeabilized cells.

### Colocalization evaluation of immunofluorescence

Colocalization of epitopes was evaluated by the image analysis carried out using the Coloc2 plugin in FIJI software calculating three different correlation coefficients as suggested in [61]. The nuclear outlines were segmented manually. The degree of colocalization was determined by Pearson’s correlation coefficient, Spearman’s rank correlation value, and Manders’ correlation coefficients (M1 and M2) of the signals from the two analyzed channels. The significance of each statistical analysis was determined by the Student’s t-tests. The randomized images were obtained as described in [61].

### Nuclear lysate preparation

One litre of suspension culture of HeLa cells was spun at 1300 g at 4°C for 15 min. The pellet was resuspended in 7 mL of buffer (50 mM Hepes pH 7.4, 150 mM NaCl, 1 mM DTT, cOmplete (La Roche Ltd., Basel, Switzerland, 05056489001)) and subjected to 20 strokes in Dounce homogenizer. Cell nuclei were sedimented by 1800 g centrifugation at 4°C for 5 min. The supernatant was collected as a cytoplasmic fraction. The nuclear pellet was washed four times in 10 mL of buffer. The clean nuclear pellet was sonicated in Soniprep 150 (MSE, London, UK) bench top sonicator (1 s on, 1 s off for 30 cycles at the power of 10 microns amplitude). Sonicated lysate was spun down at 13,000 g at 4°C for 15 min. The supernatant was collected as a nuclear fraction. Protein concentration was determined by Pierce BCA Protein Assay (Thermo Fisher Scientific, Waltham, MA, USA, 23227) according to the manufacturer’s protocol.

### Preparation of PLCδ1 PH domain

For expression and purification of GST-tagged recombinant proteins PLCδ1 PH domain (1–140 aa) wild type and R40A mutation of PLCδ1 PH domain in pGST5 were used plasmids constructed previously [62]. The PIP-binding protein domains were expressed in 100 mL of BL21 (DE3)-pLysS *E*. *coli* (Stratagene, Santa Clara, USA) culture. Transformed cells were incubated for approximately 4 h at 37°C until OD = 0.6. Expression was then induced by 0.1 mM IPTG for an additional 2 h. Samples were lysed by sonication with Soniprep 150 (MSE, London, UK) benchtop sonicator (4 s on, 4 s off for 1 min at power 10 microns of amplitude) in ice-cold buffer (50 mM Hepes pH 7.5, 150 mM NaCl, 1 mM DTT, cOmplete (La Roche Ltd., Basel, Switzerland, 05056489001)) and spun down at 13,000 g at 4°C for 15 min. Supernatants were used for purification of recombinant proteins by 2 h incubation with GST-agarose beads at 4°C according to the manufacturer’s protocol (Sigma Aldrich, St. Louis, USA, G4510). SDS-PAGE electrophoresis was used to check the level of expression and purity of purification.

### Pull-down assay with PIPs-conjugated beads at various treatment conditions

Four mL of nuclear lysate from suspension culture of HeLa cells at protein concentration of 2.5 mg/mL were prepared in buffer (50 mM HEPES, pH 7.4, 150 mM NaCl, 1 mM DTT, cOmplete (La Roche Ltd., Basel, Switzerland, 05056489001) and PhosStop RNase inhibitors (La Roche Ltd., Basel, Switzerland, 4906837001) and 2 mM MgCl_2_ for experiments “PIP2-conjugated agarose beads pull-down assays from nuclear lysates with added nuclear RNA extract upon different conditions.”, S18 and S20). The following types of agarose beads were used in pull-down assays: Control Beads, P-B000; PI(3)P beads, P-B003A; PI(4)P beads, P-B004A; PI(5)P beads, P-B005A; PI(3,4)P2 beads, P-B034A; PI(3,5)P2 beads, P-B035A; PI(4,5)P2 beads, P-B045A; PI(3,4,5)P3 beads, P-B345A (Echelon Biosciences Inc., UT, USA). Forty μL of beads slurry were added into 650 μL of nuclear lysate nuclear in experiment shown in S19 Fig supplemented with 30 μg of RNA with the increasing concentration of MgCl_2_ (0 mM, 0.1 mM, 0.5 mM, 1 mM, 2.5 mM, 5 mM, 10 mM, 25 mM, 50 mM and 100 mM) per condition and incubated overnight at 4°C. To test the biochemical nature of PIP2-BRD4 and PIP2-CAND1 association (experiment “PIP2-conjugated agarose beads pull-down assays from nuclear lysates with added nuclear RNA extract upon different conditions.” and S18), the following treatments were used in the respective specimens: the addition of 30 μg of nuclear RNA extract, 300 mM NaCl, 100 mM NH_4_OAc, 10% 1,6-hexanediol, and 10% dextran. The beads were washed three times with 1 mL of ice-cold buffer and spun down at 800 g at 4°C for 5 min. The supernatant was discarded, and the beads were boiled in 30 μL of Laemmli buffer for 10 min. The beads were spun down, and the supernatant was loaded into the SDS-PAGE gel. After trans-blotting, the membranes were blocked with 3% BSA for 30 min. The membranes were washed with 0.5% Tween 20/PBS for 15 min. The dilution of the primary antibody (Table 1) was prepared in 3% BSA/PBS and incubated for 2 h. Secondary antibody was used according to manufacturer’s instruction (Table 1). Western blot (WB) signals at each pull-down condition in every repetition were normalized to the highest signal (the PIP2 beads with RNA added condition). Statistical analysis was performed using Student’s t-tests on three replicates.

### Isolation of natural PIP2-structures and testing of BRD4-association

Four mL of nuclear lysate from suspension culture of HeLa cells at protein concentration 2.5 mg/mL were prepared in buffer (50 mM HEPES, pH 7.4, 150 mM NaCl, 2 mM MgCl_2_, 1 mM DTT, cOmplete (La Roche Ltd., Basel, Switzerland, 05056489001) and PhosStop RNase inhibitors (La Roche Ltd., Basel, Switzerland, 4906837001)). Two types of GST agarose beads were used in the pull-down assays: GST-PLCδ1 PH domain (1–140 amino acids, wild type) and R40A mutation of GST-PLCδ1 PH domain. Twenty μL of beads slurry were added into 650 μL of nuclear lysate per condition and incubated overnight at 4°C. To test the biochemical nature of BRD4 association with PIP2-containing structures, the following treatments were used in respective specimens in experiments “PIP2-conjugated agarose beads pull-down assays from nuclear lysates with added nuclear RNA extract upon different conditions.”: the addition of 30 μg of nuclear RNA extract, 300 mM NaCl, 100 mM NH_4_OAc, 10% 1,6-hexanediol, and 10% dextran. The beads were washed three times with 1 mL of ice-cold buffer and spun down at 800 g at 4°C for 5 min. The supernatant was discarded, and the beads were boiled in 30 μL of Laemmli buffer for 10 min. The beads were spun down, and the supernatant was loaded into the SDS-PAGE gel. After trans-blotting, the membranes were blocked with 3% BSA for 30 min. The membranes were washed by 0.5% Tween 20/PBS for 15 min. The dilution of the BRD4 primary antibody was prepared in 3% BSA/PBS and incubated for 2 h. A secondary antibody was used according to the manufacturer’s instructions (Table 1). WB signals at each pull-down condition in every repetition were normalized to the highest signal of the wild type GST-PLCδ1 PH domain with RNA added condition. Statistical analysis was performed using Student’s t-tests on six replicates.

### PIP2-conjugated beads pull-down assay with spiked recombinant GST-PLCδ1 PH domain

Two mL of nuclear lysate from suspension culture of HeLa cells at protein concentration of 2.5 mg/mL were prepared in buffer (50 mM HEPES, pH 7.4, 150 mM NaCl, 2 mM MgCl_2_, 1 mM DTT, cOmplete (La Roche Ltd., Basel, Switzerland,05056489001) and PhosStop RNase inhibitors (La Roche Ltd., Basel, Switzerland, 4906837001)). Two types of agarose beads were used in pull-down assays: control beads, P-B000 (Echelon Biosciences Inc., UT, USA) and PI(4,5)P2-conjugated beads, P-B045A (Echelon Biosciences Inc., UT, USA). Forty μL of beads slurry were added into 650 μL of nuclear lysate per condition and incubated overnight at 4°C. To test the specificity of the effect of RNA on PIP2 binding of BRD4 protein the addition of 30 μg of nuclear RNA extract and 1 μg of purified recombinant soluble GST-PLCδ1 PH domain (1–140 amino acids, wild type or R40A mutation), were used in the respective specimens in experiments “PIP2-conjugated agarose beads pull-down assays from nuclear lysates with added nuclear RNA extract upon different conditions.”. The beads were washed three times with 1 mL of ice-cold buffer and spun down at 800 g at 4°C for 5 min. The supernatant was discarded, and the beads were boiled in 30 μL of Laemmli buffer for 10 min. The beads were spun down, and the supernatant was loaded into the SDS-PAGE gel. After trans-blotting, the membranes were blocked with 3% BSA for 30 min. The membranes were washed with 0.5% Tween 20/PBS for 15 min. The dilutions of the primary antibodies (anti-GST, anti-BRD4) were prepared in 3% BSA/PBS and incubated for 2 h. Secondary antibodies were used according to the manufacturer’s instructions (Table 1). WB signals at each pull-down condition in every repetition were normalized to the signal from the PIP2-beads pull-down signal, wild-type GST-PLCδ1 PH domain–the condition with RNA added. Statistical analysis was performed using Student’s t-tests on four replicates.

### Mass spectrometry experimental pipeline

One mL of nuclear fraction from suspension culture of HeLa cells with a protein concentration of 2.5 mg/mL was used per condition in three independent biological replicates. Twenty-five μL of washed wild-type or R40A mutation GST-PLCδ1 PH domain immobilized to glutathione agarose beads were added into the respective reactions and incubated at 4°C for 1.5 h while rotating to allow PIP2–PH domain interaction. The RNase III-treated samples were pre-incubated with 4 U of RNase III (New England Biolabs, Ipswich, Massachusetts, USA, M0245S) in a reaction supplemented by 20 mM MnCl_2_ at 15°C for one hour. These samples were incubated with wild-type GST-PLCδ1 PH domain. After the incubation of all samples, the beads were centrifuged at 300 g at 4°C for 2 min. The supernatant from each sample was carefully discarded. Beads were spun down and washed twice in 1 mL of buffer (50 mM Hepes, pH 7.4, 150 mM NaCl, 1 mM DTT, cOmplete (La Roche Ltd., Basel, Switzerland, 05056489001)) and subjected to sample preparation for MS measurement. Beads were resuspended in 100 mM triethylammonium bicarbonate (TEAB) containing 2% sodium deoxycholate (SDC). Proteins were eluted and cysteines were reduced in one step by heating with 10 mM final concentration of Tris-(2-carboxyethyl)phosphine (TCEP; 60°C for 30 min). Beads were removed by centrifugation, and proteins in the supernatant were incubated with a 10 mM final concentration of methyl methanethiosulfonate (MMTS; 10 min RT) to modify reduced cysteine residues. In-solution digestion was performed with 1 μg of trypsin at 37°C overnight. After digestion, the samples were centrifuged, and the supernatants were collected and acidified with trifluoroacetic acid (TFA, final concentration of 1%). SDC was removed by ethylacetate extraction [63]. Peptides were desalted using homemade stage tips packed with C18 disks (Empore) according to Rappsilber et al. [64].

One liter of suspension culture of HeLa cells was spun at 1300 g at 4°C for 15 min. The pellet was resuspended in 7 mL of buffer (50 mM Hepes pH 7.4, 150 mM NaCl, 1 mM DTT, cOmplete (La Roche Ltd., Basel, Switzerland, 05056489001)) and subjected to 20 strokes in Dounce homogenizer. Cell nuclei were sedimented by 1800 g centrifugation at 4°C for 5 min. The supernatant was collected as a cytoplasmic fraction. The nuclear pellet was washed four times in 10 mL of buffer. The clean nuclear pellet was sonicated in Soniprep 150 (MSE, London, UK) bench top sonicator (1 s on, 1 s off for 30 cycles at the power of 10 microns amplitude). Sonicated lysate was spun down at 13,000 g at 4°C for 15 min. The supernatant was collected as a nuclear fraction. Three independent biological replicates per sample were prepared for MS analysis as described above.

### Liquid chromatography-tandem mass spectrometry (LC-MS/MS) analysis

Nano reversed-phase column (EASY Spray column, 50 cm, 75 μm ID, PepMap C18, 2 μm particles, 100 Å pore size) was used for LC-MS/MS analysis. The mobile phase A was composed of water and 0.1% formic acid. The mobile phase B was composed of acetonitrile and 0.1% formic acid. The samples were loaded onto the trap column (Acclaim PepMap 300, C18, 5 μm, 300 Å, 300 μm, 5 mm) at 15 μL/min for 4 min. The loading buffer was composed of water, 2% acetonitrile, and 0.1% TFA. Peptides were eluted with the mobile phase B gradient from 4% to 35% in 60 min. Eluting peptide cations were converted to gas phase ions by electrospray ionization and analyzed on a Thermo Orbitrap Fusion (Q OT qIT, Thermo Fisher Scientific, Waltham, MA, USA). Survey scans of peptide precursors from 350 to 1400 m/z were performed at 120 K resolution (at 200 m/z) with a 5x10^5^ ion count target. Tandem MS was performed by isolation at 1.5 Th with the quadrupole, HCD fragmentation with normalized collision energy of 30, and rapid scan MS analysis in the ion trap. The MS/MS ion count target was set to 10^4^, and the max injection time was 35 ms. Only those precursors with charge states 2–6 were sampled for MS/MS. The dynamic exclusion duration was set to 45 s with a 10 ppm tolerance around the selected precursor and its isotopes. Monoisotopic precursor selection was turned on. The instrument was run in top speed mode with 2 s cycles [65].

### Raw data processing

Raw data files acquired by LC-MS/MS were processed with MaxQuant v1.6.11.0 [66]. Peak lists were searched against the human SwissProt database (May 2020) using the Andromeda search engine [67]. The minimum peptide length was set to seven amino acids, and two missed cleavages were allowed. Dithiomethylation of cysteine was set as a fixed modification, while oxidation of methionine and protein N-terminal acetylation were used as variable modifications. Only peptides and proteins with a false discovery rate (FDR) lower than 0.01 were accepted. Protein intensities were normalized using MaxLFQ algorithm [68]. MaxQuant output data were further analyzed using Perseus v1.6.13.0 [69] and visualized in R v4.0.0 [70]. Briefly, protein groups identified at the 0.01 FDR level were further filtered to remove potential contaminants, decoys, and proteins identified based on modified peptides only. The resulting matrix was filtered based on the number of missing values (100% of valid values in at least one of the groups), and after log2 transformation, missing values were imputed from a normal distribution (width = 0.3 times standard deviation (SD) and shift = 1.8 times SD of the original distribution). One replicate from the samples enriched using the wild-type GST-PLCδ1 PH domain-conjugated beads was identified as an outlier with an overall lower MS intensity and removed from the downstream analysis.

### Identification of nuclear RNA-dependent PIP2-associated (RDPA) proteins

A two-step statistical analysis was performed to identify the RNA-dependent PIP2-associated (RDPA) proteins. In the first step, the “PIP2-associated proteome” was identified by comparing the proteome enriched using the wild-type GST-PLCδ1 PH domain-conjugated beads to the proteome enriched using the point mutation (R40A) GST-PLCδ1 PH domain-conjugated beads; a one-sided Student’s t-test was performed with a permutation-based FDR correction. In the second step, the significant proteins (FDR < 0.05, S0 = 0.2, n = 195 protein groups) were then compared to the samples enriched using the wild-type PLCδ1 PH domain-conjugated beads from samples pretreated with RNase III. A two-sided Student’s t-test with a permutation-based FDR correction was applied to identify differentially enriched (FDR < 0.05, S0 = 0.2) protein groups after RNase III treatment (n = 183; downregulated n = 168). In both comparisons, Student’s t-test was performed in Perseus v1.6.13.0 (S1 Table). The results were visualized using the R package “ggplot2”.

### Manipulation of PIP2 level in cells

MISSION esiRNA (Sigma-Aldrich, USA, EHU114801-20UG) was used to deplete human PIP5K1A. MISSION esiRNA (Sigma-Aldrich, USA, EHU081051-20UG) was used for depletion of the human SHIP2. MISSION siRNA Universal Negative Control #1 (Merck, NJ, USA, SIC001) was used as the negative control. U2OS cells were seeded 24 h before the transfection on 12 mm in diameter glass coverslips with restricted thickness-related tolerance (depth = 0.17 mm ± 0.005 mm) and the refractive index = 1.5255 ± 0.0015 (Marienfeld, 0107222) at 70% confluency. The cells were transfected using Lipofectamine RNAiMax (Invitrogen, MA, USA, 13778150) for 24 h according to the manufacturer’s protocol and subjected to immunofluorescence staining. Protein depletion efficiency was confirmed by WB assay (S21B Fig) and quantification of changes in nuclear PIP2 levels was performed based on the IF signal. Alternatively, the SHIP2 inhibitor K149 at a concentration of 10 μM (Echelon Biosciences Inc., UT, USA, B-0345) was used for 1 hour to increase PIP2 levels (S22 Fig). The images were acquired at Leica STELLARIS 8 FALCON (Leica Mikrosysteme Vertrieb GmbH, Wetzlar, Germany) confocal microscope with × 63 oil objective NA 1.4.

### Image analysis of BRD4 protein foci

Identification of foci was performed using a macro pipeline written in FIJI software [60]. Briefly, the channels from the Z-stack acquisition were split and analyzed in sequence. First, the nuclear area detected by the DAPI signal was identified. The nuclear area was further processed using the 3D Gaussian blur function. Next, the channel with visualized protein was processed by Gaussian blur 3D on the ROI previously identified as the nuclear area and the outside was deleted. The 3D object counter was then used to identify the protein foci using a minimum size filter of 10 voxels. The results showed the number of foci per cell. Statistical analysis was performed using the Student’s test.

### Functional characterization of the RDPA proteome using Metascape

The functional gene ontology (GO) analysis of the RDPA proteome (proteins associated with PIP2 in RNA-dependent positive manner) was performed by the Metascape tool using the default settings [71]. The protein list for Metascape analysis comprised of 168 proteins; one majority protein ID was selected per protein group (S17 Table). The analysis was performed against a default Metascape background set—human proteome (S18 Table), or against proteins identified in the nucleus—Nuclear fraction proteins (183 RDPA proteins were analyzed, with all majority protein IDs selected per protein group; S19 and S20 Tables). The enriched functional terms were identified using a default Metascape algorithm using a hypergeometric test. The significant terms were then hierarchically clustered into a tree based on Kappa-statistical similarities among their gene memberships. Then 0.3 kappa score was applied as the threshold to cast the tree into term clusters. We then selected a subset of representative terms from this cluster and converted them into a network layout. More specifically, each term is represented by a circle node, where its size is proportional to the number of input genes belonging to the term, and its color represents its cluster identity (i.e., nodes of the same color belong to the same cluster). Terms with a similarity score > 0.3 are linked by an edge (the thickness of the edge represents the similarity score). The network was visualized with Cytoscape (v3.1.2) applying a “force-directed” layout. One term from each cluster was selected to have its term description shown as a label.

### Data preparation for bioinformatic analyses

Majority protein IDs from significant protein groups (see Raw data processing) were mapped to the UniProtKB database [72] (*Homo sapiens*, Swiss-Prot, reference proteome UP000005640, release 2022_01) and their canonical protein sequences were obtained. Seven datasets were used for further analyses: i) proteins associated with PIP2 in higher-order RNA-dependent positive manner (called RDPA proteins, n = 183); ii) proteins quantifiable in at least two replicates of nuclear fraction, but not in cytosolic fraction, with RDPA proteins added (called Nucleo-specific proteins, n = 848); iii) proteins quantifiable in at least two replicates of nuclear fraction, with RDPA proteins added (called Nuclear fraction proteins, n = 3,655); iv) proteins quantifiable in at least two replicates of cytosolic fraction, but not in nuclear fraction (called Cytosol-specific proteins, n = 428); v) proteins quantifiable in at least two replicates of cytosolic fraction (called Cytosolic fraction proteins, n = 3,379); vi) all proteins quantifiable in at least two replicates of cytosolic or nuclear fraction, with RDPA proteins added (called Total cell proteome, n = 4,082) and vii) reference human proteome from UniProtKB with one protein sequence per gene (called Reference proteome, n = 20,577). The complete list of employed protein IDs and the graphical illustration of overlaps between original datasets are in S3 Fig and S3 and S4 Tables.

### Search for protein domains binding to PIP2

Protein domains able to bind to PIP2 were selected based on previously published data [73–76]. The PROSITE database [77] and InterPro database [78] were used to search for proteins with such features in the Swiss-Prot database (*Homo sapiens*, reference proteome UP000005640, release 2022_02), resulting in 1,552 distinct proteins. This protein list was then compared with our datasets (S5 Table).

### RNA-binding capability

Protein IDs from datasets were compared to the list of all human proteins with experimental evidence for RNA-binding, according to the RNAct database [79] (3,717 reviewed proteins from Swiss-Prot, mapped to the reference proteome UP000005640) (S6 and S8 Tables).

### Association with phase separation

Protein IDs from datasets were compared to the list of all human proteins associated with phase separation or membraneless organelles, according to PhaSepDB2.0 [80] (4,014 reviewed proteins from Swiss-Prot, mapped to the reference proteome UP000005640) (S7 and S8 Tables).

### Prediction of intrinsically disordered regions (IDRs)

Disordered regions were predicted by ESpritz (version 1.3) [81] with three prediction types: X-Ray, Disprot, and NMR, and a decision threshold of 5% False Positive Rate (S9 Table). For further analysis, an R-based script (R, version 4.3.1) [70] was created, which uses UniProtKB accession numbers and searches them against the Database of Disordered Protein Predictions (D2P2) [82]. However, not all UniProtKB accession numbers were successfully mapped to D2P2. On average, we retrieved the information for 95.6% (from 94.5% to 96.5%) of the input protein sequences for most of the datasets. The exception was the Reference proteome, where we retrieved information for 88.9% of the input protein sequences. Protein was counted as IDR-containing protein only if at least one IDR with a minimum length of 20 amino acid residues was predicted in this protein. The pI and hydrophobicity of the disordered regions were calculated using the R package “peptides”, functions pI, and hydrophobicity. The hydrophobicity of each IDR was determined based on grand average of hydropathy (GRAVY) value, calculated as the sum of hydropathy values of all amino acids in the IDR divided by the length of the IDR. Thus, lower values indicate more hydrophilicity and higher values more hydrophobicity of an IDR.

### K/R-rich motifs abundance analysis

Short sequence motifs rich for lysine and/or arginine (K/R motifs) were analyzed as described previously [83]. Briefly, K/R motifs: [KR]-x(3,7)-K-x-[KR]-[KR], [KR]-x(3,7)-K-x-[KR] and [KR]-x(3,7)-K-x-K were searched in all datasets using ScanProsite tool [84], match mode set as greedy, no overlaps (S10 Table).

### Analysis of K/R motifs enrichment in IDRs

The analysis was performed using a custom R-based script (R, version 4.3.1), which uses information about the presence of K/R motif in the dataset from the ScanProsite tool, and searches UniProtKB accession numbers of such proteins against D2P2. K/R motif was counted as present in IDR only if at least three predictors from the D2P2 predicted IDR with a minimum length of 20 amino acid residues at the site of the K/R motif (S11 Table).

### Analysis of the function of K/R motifs in IDRs

Localization of K/R motifs (i.e., [KR].{3,7}K.[KR][KR], [KR].{3,7}K.[KR] and [KR].{3,7}K.K) in proteins to structured regions or IDRs and GO analyses of proteins containing such motifs were assessed with SLiMSearch4 tool [85], with disorder score cut-off set to 0.95 (S21–S29 Tables). The results of the analysis were visualized using “bubble plots”. In the plots, the y-axis shows the–log10 adjusted p-value of proteins from a GO category, and the x-axis shows the log2 enrichment factor. The size of the bubble corresponds to the number of proteins.

### PTM site proximity analysis

A database of known posttranslational modification (PTM) sites was downloaded (August 6, 2022) from the PhosphoSitePlus database [86]. For the following known PTMs: acetylation, methylation, phosphorylation, sumoylation, and ubiquitination, we explored whether they are located in the IDR containing the K/R motifs identified by the abovementioned analysis of K/R motifs enrichment in IDRs using a custom R-based script (S16 Table).

### Statistical analyses and data visualization

Statistical relevance of depletion or enrichment of a particular feature (e.g., IDR content) between datasets was analyzed in R, version 4.1.3 [70] using a hypergeometric test (function phyper). All plots were generated using the R package “ggplot2” [87]. In the boxplots, the bold line indicates the median value; box borders represent the 25th and 75th percentiles, and the whiskers represent the minimum and maximum value within 1.5 times of interquartile range. Outliers out of this range are depicted using solid dots.

## Results and discussion

### RNA is important for PIP2 nuclear localization

**Fig 1:**
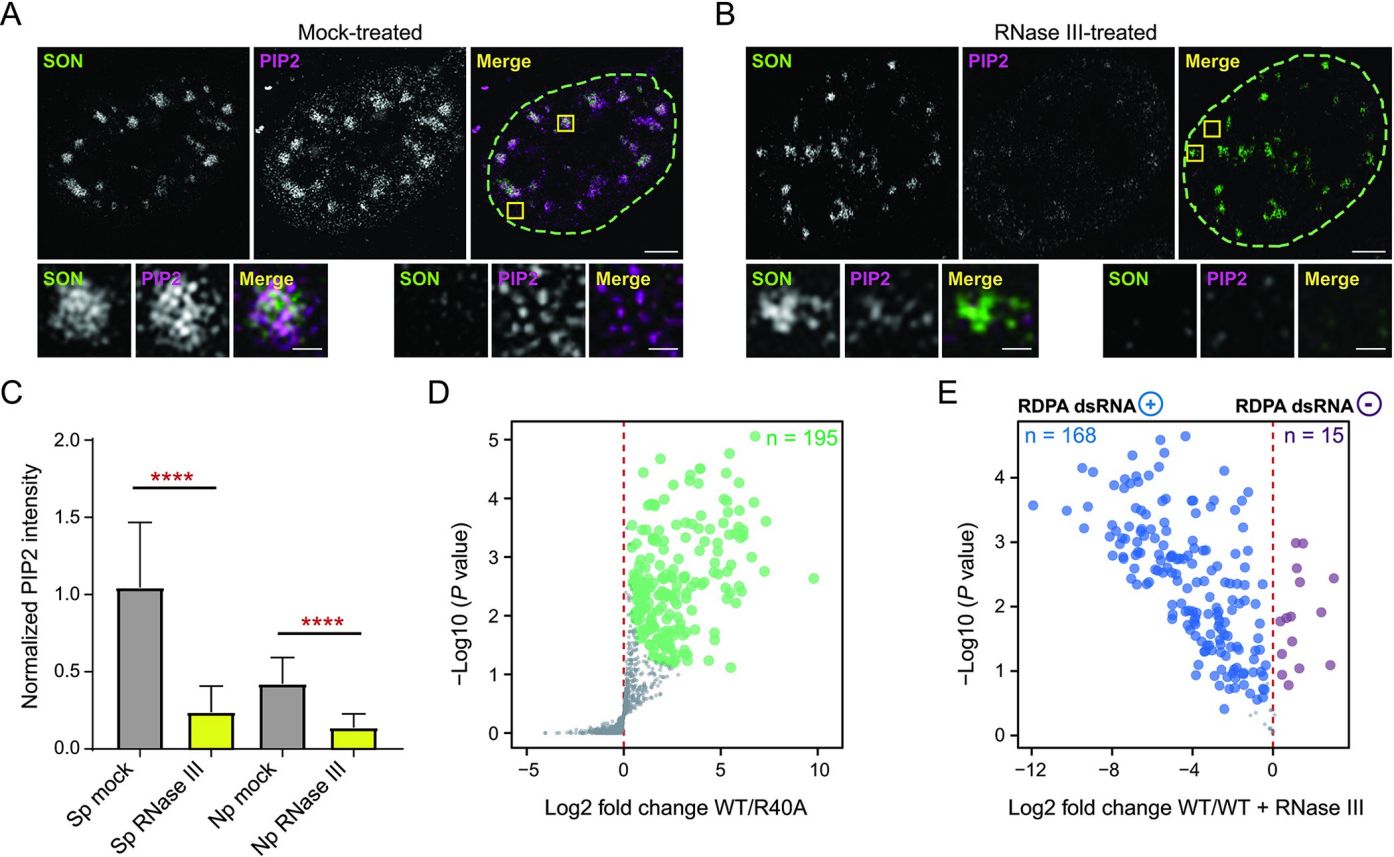
Identification of the RNA-dependent PIP2-associated (RDPA) nuclear proteome. **(A-B)** The effect of RNase III treatment on PIP2 and SON localization in cell nuclei visualized by immunofluorescence staining and super-resolution microscopy. U2OS cells were semi-permeabilized, treated by RNase III, and subsequently stained with PIP2 and SON-specific antibodies. Images were acquired by structured illumination microscopy (SIM). **A)** Mock-treated U2OS cell nucleus, **B)** RNase III-treated nucleus with detailed zoom-in insets of PIP2 and SON localization in the nucleoplasmic and nuclear speckle subcompartments. Scale bars correspond to 5 μm and 1 μm in the image and inset details, respectively. **C)** Quantification of normalized mean PIP2 signal intensity levels upon RNase III treatment in segmented nuclei for nuclear speckles (Sp) and nucleoplasm (Np) regions. Statistical analysis was performed using Student’s t-tests. Error bars correspond to SEM (**** P < 0.0001), n = 3, N = 76 cells mock-treated, N = 89 RNase III-treated cells. **D)** Volcano plot shows the PIP2-associated nuclear proteome identified by MS analysis; the significantly enriched protein groups (n = 195) are depicted in green (FDR < 0.05 & S0 = 0.2). **E)** Volcano plot of proteins whose PIP2-structures association is regulated by the presence of higher-order RNA. Protein groups showing a statistically significant (FDR < 0.05 & S0 = 0.2) loss (n = 168; RDPA dsRNA+, marked in blue, positively regulated by higher-order RNA) or gain (n = 15; RDPA dsRNA–, marked in purple, negatively regulated by higher-order RNA) of association with PIP2 after dsRNA cleavage are shown in blue or purple, respectively. A Student’s t-test with a permutation-based FDR correction was performed using a function provided in the Perseus software.

RNA is the critical integral element for the coherence of many membraneless structures [88,89]. Indeed, RNA is important in regulating the phase separation of proteins forming condensate assemblies [90]. The scaffolding RNA typically adopts higher-order folds that are enabled by the formation of dsRNA regions [91,92]. We therefore hypothesized that higher-order RNA structures are important for PIP2 localization in the nucleus. To test this hypothesis, we used RNase III treatment of semi-permeabilized cells followed by immunofluorescence labeling of PIP2 and the nuclear speckles marker protein SON. RNase III does not have a conserved target sequence but recognizes dsRNA structures [93,94]. The nucleus is a very dense environment and nuclear PIP2 forms sub-diffraction-limited foci in the nucleoplasm, so we used super-resolution microscopy [95–98]. We observed that RNase III-mediated RNA cleavage greatly reduced the total nuclear PIP2 signal, whereas based on SON staining, the structural integrity of nuclear speckles is not completely abolished (Fig 1A and 1B). In addition, our results show that PIP2 levels in both the nucleoplasm and nuclear speckles decrease to a similar extent upon RNA cleavage (Fig 1C). The RNase III treatment of non-permeabilized cells had no effect on nuclear PIP2 levels (S1 Fig). These results suggest that the nuclear localization of PIP2 is dependent on the presence of higher-order RNA in specific regions. Thus, this observation postulates an intimate relationship between PIP2 and RNA in the nucleus.

### Identification of RNA-dependent PIP2-associated nuclear proteome

We searched for proteins that are important for the formation of the nuclear architecture dependent on the interplay between RNA and PIP2. Given the importance of RNA in the formation of nuclear subcompartments, it has been suggested to use RNases to identify proteins involved in the formation of nuclear structures generated by phase separation [92]. We aimed to identify the proteins, presumably components of complexes that associate with PIP2-containing structures in a higher-order RNA-dependent manner. The phospholipase C pleckstrin homology (GST-PLCδ1 PH) domain has a well-documented specificity for PIP2 binding. Therefore, we developed a label-free quantitative MS approach based on GST-PLCδ1 PH domain pull-downs from nuclear lysates treated or untreated with RNase III. The wild-type GST-PLCδ1 PH domain binds PIP2 with high specificity, whereas its point mutation R40A abolishes its binding [62]. We confirmed the specificity of GST-PLCδ1 PH domain to bind PIP2 by labeling its purified recombinant GST-tagged proteins combined with PIP2-specific antibody in immunolabeling of U2OS analyzed by confocal microscopy. Both the antibody and the recombinant GST-PLCδ1 PH domain show a high degree of signal overlap (S2 Fig). In the first step of this experimental workflow, we prepared nuclear lysates suitable for comparative MS as described previously [83]. The wild-type and R40A GST-PLCδ1 PH domain variants were attached to agarose beads via a glutathione S-transferase (GST) tag and incubated with the nuclear lysates. Analysis of MS data of proteins bound to both domains with differential abundance led to the identification of the PIP2-associated nuclear proteome (Fig 1D). In parallel, dsRNA in the third nuclear lysate sample was digested with RNase III prior to the wild-type GST-PLCδ1 PH domain pull-down and subsequent MS measurement. This step resulted in the depletion of 168 protein groups and the enrichment of 15 protein groups, allowing us to identify PIP2-associated proteins that were differentially changed upon dsRNA cleavage (Fig 1E). The approach presented here identifies not only direct but also indirect interactors, i.e., components of protein complexes. It is a different approach than using immobilized PIP2 on beads, which we have previously used to identify PIP2 interactors [83]. Thus, the GST-PLCδ1 PH domain-based approach identifies naturally occurring PIP2-containing nuclear structures associated with protein complexes. Therefore, this experimental pipeline allowed us to determine the nuclear RNA-dependent PIP2-associated (RDPA) proteome. Our data show that the vast majority of PIP2-associated proteins lose their PIP2 association upon removal of higher-order RNA (Fig 1E).

PIP2 is normally embedded in cytoplasmic membranes where it regulates various processes through interactions with a variety of proteins with PIPs binding domains. We hypothesized that nuclear PIP2 might be also involved in the recognition and binding of various proteins, thereby regulating the localization of their actions. Such proteins typically contain canonical PIPs binding domains with well-defined globular structures, e.g., pleckstrin homology (PH), phox homology (PX), Fab-1, YGL023, Vps27 and EEA1 (FYVE) domains, etc. To assess the abundance of PIPs binding domains in the RDPA proteome, we generated in parallel several reference datasets by analyzing the nuclear and cytosolic fractions isolated from the same HeLa cell line using the same LC-MS/MS proteomic pipeline (see M&M section for more details). We then searched for these domains in the RDPA proteome and in datasets of proteins identified exclusively in the nucleus (’nucleo-specific’, 848 proteins) and in the cytosol (’cytosol-specific’, 428 proteins). In addition, we generated the ’total cell proteome’ (4,082 proteins) by combining all proteins identified in our MS analyses of nuclear and/or cytosolic proteomes. For comparison, we also provide the analysis of the additional proteomes, i.e., ’nuclear fraction’ proteins, ’cytosolic fraction’ proteins, and ’reference proteome’ (S3 Fig and S3 and S4 Tables; see M&M for details). However, in agreement with previously published data [2,83], the frequency of canonical PIPs binding domains was very low in both RDPA subpopulations. Only 11 out of 183 RDPA proteins positively regulated by the presence of higher-order RNA contain the PIPs binding domains (S5 Table), suggesting that these domains are not a major route of PIP2 association. Interestingly, none of the proteins negatively regulated by the presence of higher-order RNA possess such a domain. Notably, this dataset (16 proteins) that showed increased PIP2 binding upon higher-order RNA cleavage is rather small for reasonable statistical correlation with other datasets. Therefore, we focused further analyses only on RDPA proteins with a positive higher-order RNA effect on their PIP2 binding, and unless otherwise noted, the term RDPA proteins refers to this subpopulation.

### RDPA proteome is enriched for proteins with phase separation capacity and PIP2-binding motifs in their IDRs

**Fig 2:**
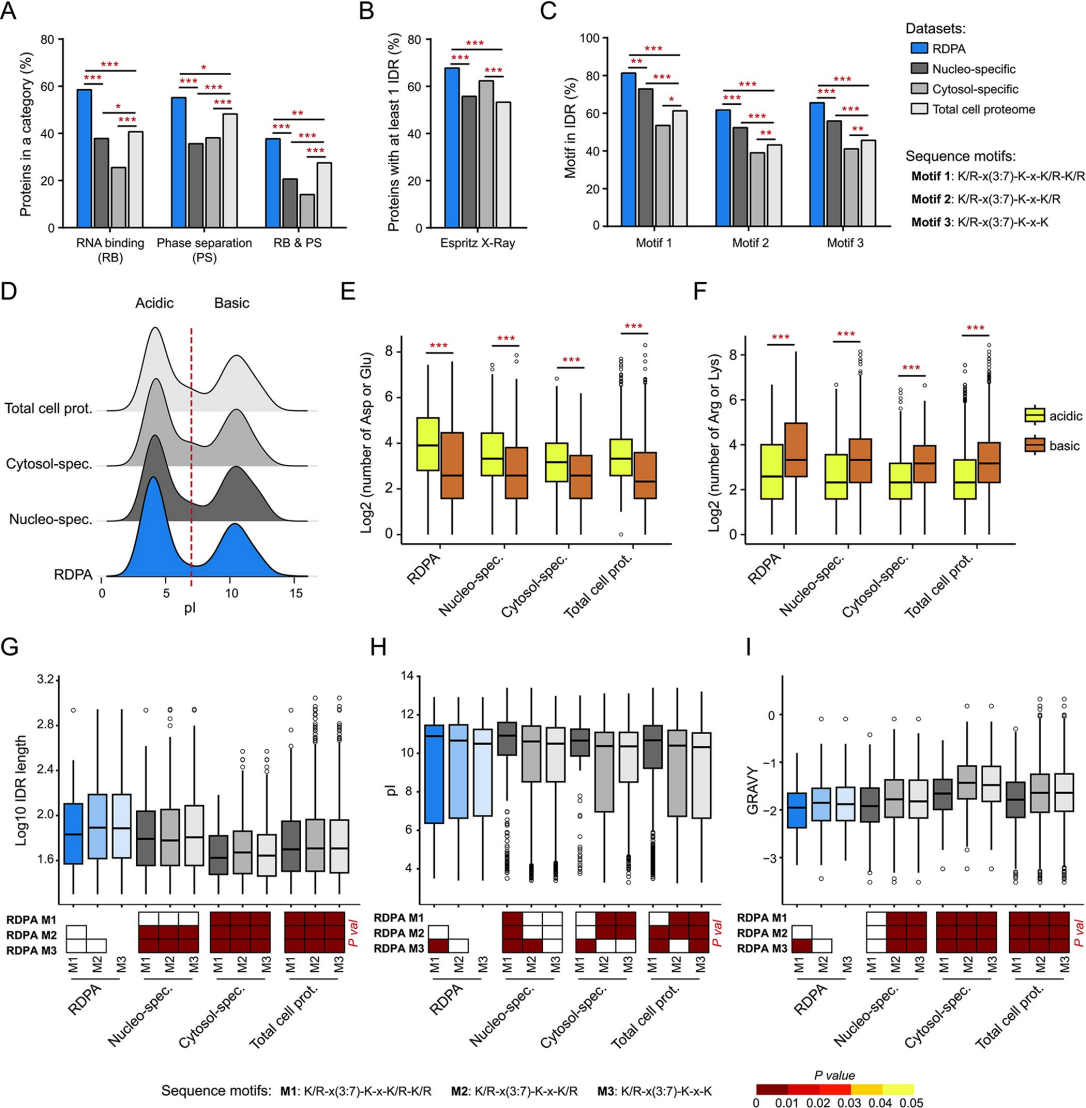
Bioinformatic analyses of RDPA proteome features. **A)** RDPA proteome is significantly enriched for RNA binding, phase separation capacity, and combination of both properties. **B)** RDPA proteome is enriched for IDRs longer than 30 amino acid residues predicted by ESpritz X-Ray. **C)** Percentage of PIP2-binding K/R motif sites localized in IDRs (from all K/R motif sites in the dataset) is elevated in RDPA proteome (only IDRs predicted by at least three different predictors with minimal length 20 amino acid residues were considered). Statistical analysis was performed using a hypergeometric test (* P < 0.05, ** P < 0.01, and *** P < 0.001). **D)** pI values of IDRs predicted by ESpritz X-Ray in the analyzed protein datasets show a bimodal distribution. **E)** The IDRs in the “acidic” population (pI < 7) are enriched with D/E amino acid residues. **F)** The IDRs in the “basic” population (pI > 7) are K/R-rich. Statistical analysis was performed using a Wilcox test (*** P < 0.001). **G-I)** IDRs in the RDPA proteome containing the three K/R motifs tend to be significantly longer **(G)**, more basic **(H)**, and more hydrophilic **(I)** compared to IDRs in other datasets. Statistical analysis was performed using a pairwise Wilcox test with Benjamini-Hochberg correction. Datasets: RDPA–proteins with positive higher-order RNA effect on their PIP2 binding (dsRNA+), Nucleo-specific–proteins identified exclusively in the nucleus, Cytosol-specific–proteins identified exclusively in the cytosol, and Total cell proteome–all proteins identified in the nucleus and/or cytosol.

Nuclear PIP2 localizes to two archetypal liquid-like structures formed by phase separation—nuclear speckles and nucleoli [17,25,29]. The appropriate subcompartmentalized nature of such multiphase structures depends on the presence of architectural RNAs [27,55,91,99,100]. Furthermore, there is a higher concentration of RNA in the nucleus compared to the cytosol, suggesting that RNA is the critical driving force for the preferential formation of such structures in the nucleus. Our bioinformatic analyses confirmed the enrichment of RNA-binding properties within the RDPA proteome (Figs 2A and S4A and S6 Table), a predictable feature due to the RNase III treatment step in our MS workflow. Importantly, we showed that the RDPA proteome is the most enriched for proteins with the ability to phase separate from all datasets (Figs 2A and S4A and S7 Table). This search was based on the overlap of proteins from the RDPA proteome with the PhaSEP database, which contains proteins associated with phase separation and membraneless organelles [101]. Finally, the RDPA proteome is enriched for proteins with both RNA binding and phase separation capabilities together (Figs 2A and S4A and S8 Table), suggesting that these two properties are linked, as described elsewhere [43].

Phase separation is often mediated by multivalent interactions between proteins and RNA [39,42,90]. One of the typical structural features with phase separation capacity are IDRs. It has been shown that the nuclear proteome is enriched for proteins containing IDRs [102–104], suggesting that nuclear proteins are prone to phase separation and thus to the formation of biomolecular condensates. Indeed, our bioinformatic analysis revealed that the RDPA proteome is significantly enriched for proteins containing IDRs among other datasets (Fig 2B and S9 Table). These results were confirmed using three different IDR predictors, which yielded similar results (S4B and S4C Fig).

Previously described K/R motifs have been identified as regions important for PIP2 interaction [2,83]. We therefore screened our datasets for the presence of three known K/R motifs—K/R-x(3,7)-K-x-K/R-K/R, K/R-x(3,7)-K-x-K/R and K/R-x(3,7)-K-x-K. These motifs were abundant in the RDPA proteome, but only the K/R-x(3,7)-K-x-K/R-K/R motif (the longest one) was significantly enriched compared to all other datasets (S5A and S5B Fig and S10 Table). Since the RDPA proteome is enriched for K/R motifs and RNA-binding proteins, and its PIP2 association is RNA-dependent, it can be assumed that not all RDPA proteins interact directly with PIP2. It is therefore possible that positively charged K/R motifs serve as binding sites for negatively charged RNA molecules. Different K/R motif lengths suggest localization to different nuclear loci and thus involvement in different processes. This effect is likely to be manifested by different K/R content providing different affinity and thus retention time in a particular nuclear compartment as shown elsewhere [33].

We then investigated whether these K/R motifs are localized in proteins inside or outside the predicted IDRs. In the case of the RDPA proteome, all three motifs had a significantly increased abundance in IDRs (Figs 2C and S5C and S11 Table). Interestingly, the longest, and thus least permissive motif to search, showed the highest frequency in IDRs (four times higher than outside IDRs). Based on the above data, the RDPA proteins contain PIP2-binding K/R motifs within their IDRs and possess phase separation and RNA binding capacity.

### RDPA proteins contain long hydrophilic IDRs with acidic D/E-rich and basic K/R-rich regions

The aforementioned K/R motifs within IDRs are typical examples of multivalent interaction modules employed in the phase separation-driven formation of biomolecular condensates [33–35]. To determine the distribution of net charge in the IDRs, we analyzed the isoelectric points (pI) of all predicted IDRs in the RDPA proteome. We used the Database of Disordered Protein Predictions [82], and all nine different IDR predictors showed a similar bimodal distribution pattern of IDRs (only IDRs with a minimum length of 20 amino acid residues were considered) based on their pI (Figs 2D, S6 and S7A). The acidic peak (pI < 7) represents IDRs enriched in D/E amino acid residues (Figs 2E, S7B, S8A and S8C). The second peak represents basic IDRs (pI > 7) enriched for K/R amino acid residues (Figs 2F, S7C, S8B and S8D). These data show that IDRs have a bimodal pI distribution regardless of their cell fraction origin. Interestingly, the D/E motif has been described as important for the negative regulation of protein condensation capacity [105,106]. Thus, PIP2-mediated recruitment of RDPA IDRs containing the D/E-rich regions could have a negative effect on condensation and limit the size of condensates formed. Therefore, PIP2 may function in defining the local concentration of nuclear RDPA proteins and regulating their localization and condensation capacity as suggested in [59].

We demonstrated that K/R motifs are more abundant within predicted IDRs than in external structured regions (Fig 2C). Therefore, we focused our further analysis on IDRs (predicted by at least three different predictors with a minimum length of 20 amino acid residues) containing the K/R motifs. In particular, we evaluated the average length of K/R motif-containing IDRs across different datasets. The results show that RDPA proteins have significantly longer IDRs than other datasets, regardless of the K/R motif type analyzed (Figs 2G and S9–S11 and S12 Table). Importantly, the RDPA and nucleo-specific proteomes are specifically significantly enriched for two IDR types with average lengths of ~300 and ~800 amino acid residues (S11B Fig and S13 Table). Longer IDRs are more prone to condensation due to an increased degree of intrinsic disorder [107], i.e., a higher number of disordered amino acid residues (S12 Fig and S9 Table).

Analyzing the pI distribution of K/R motif-containing IDRs confirmed the bimodal distribution of pI found in IDRs (Fig 2D), irrespective of the K/R motif present (S13B Fig). However, the pI of K/R motif-containing IDRs is more basic than acidic (S13A Fig), consistent with the higher abundance of K and R amino acid residues in basic IDRs (Fig 2F). As expected, IDRs containing the longest K/R motif have a significantly higher average pI than IDRs with shorter K/R motifs (Figs 2H, S13A and S13C and S14 Table). Furthermore, we analyzed the hydrophobicity of these IDRs using grand average of hydropathicity (GRAVY) calculations (Figs 2I, S14 and S15A and S15 Table). All datasets have hydrophilic IDRs with mean GRAVY values between -1.4 and -2.0, irrespective of the particular K/R motif, consistent with the hydrophilic nature of IDRs in general [108–110]. Next, we evaluated the GRAVY distribution between the datasets and each of the three K/R motifs (Figs 2I and S15B). The RDPA proteome possesses K/R motif-containing IDRs with a significantly higher average hydrophilicity compared to other datasets, except for the nucleo-specific proteome with the longest K/R motif. Furthermore, IDRs with the longest K/R motif are significantly more hydrophilic than IDRs with shorter K/R motifs, regardless of the dataset (S15A and S15C Fig and S15 Table). We suggest that the multimodal distribution of GRAVY of the RDPA and cytosol-specific proteomes (S15B Fig) is caused by lower protein counts in these datasets.

In summary, these data show that RDPA proteins specifically contain longer IDRs with more charged amino acid residues than other datasets. This is consistent with the previous observation that PIP2 nuclear effectors associate with charged inositol headgroups, presumably via their hydrophilic protein regions [83].

### Analysis of the presence of post-translational modification sites in the RDPA proteome

Charge is a crucial parameter of the components of biomolecular condensates. The formation of a condensate represents a metastable state when the charge is in a desirable balance. PTMs, such as phosphorylation, often induce the collapse of this balance, ultimately leading to the dissolution of a condensate [40,45,111–113]. Therefore, we retrieved the known PTM sites from the PhosphoSitePlus database [86], namely acetylation, methylation, phosphorylation, SUMOylation, and ubiquitination, and assessed their localization in the K/R motifs containing IDRs. Indeed, phosphorylation sites were the most abundant PTM sites across all datasets and the three K/R motifs analyzed, with the highest incidence in the RDPA proteome (Tables 2 and S16). Phosphorylation is a very common PTM of IDRs, usually associated with a negative effect on the propensity for phase separation [113]. It has been shown that differential phosphorylation of the intrinsically disordered C-terminal domain of Pol2 alters its condensation capacity and integration into transcription initiation and splicing condensates [45,114].

Furthermore, RDPA proteome IDRs containing the K/R-x(3,7)-K-x-K/R-K/R motif were enriched for all screened PTM sites except SUMOylation (Table 2). On the contrary, the cytosol-specific proteins generally showed a low rate of acetylation, methylation, and SUMOylation PTM sites, which is in agreement with the literature [115–117]. Acetylation and methylation are two very common modifications of histone proteins that determine chromatin accessibility and thus the rate of transcription [118]. The subcompartmentalization of differentially active chromatin is driven by the phase separation, suggesting that these PTMs are indeed critical features that define the dynamic nuclear architecture and influence gene expression [119,120]. The above data show that PIP2-associated IDRs are sites of intense PTM regulation, suggesting their importance in regulating processes that depend on protein condensation capacity.

**Table 2:** Frequency of posttranslational modification sites within IDRs containing K/R motif.

Dataset	#	% of IDRs containing K/R-x(3,7)-K-x-K/R-K/R motif and PTM(s)				
		Acetylation	Methylation	Phosphorylation	Ubiquitination	SUMOylation
**RDPA proteome**	126	43.65	21.43	90.48	46.83	5.56
**Nucleo-specific**	349	36.68	20.34	82.23	34.38	9.74
**Cytosol-specific**	107	19.63	7.48	71.03	42.06	0.93
**Total cell proteome**	1225	35.76	19.27	79.27	46.45	12.08

#—Total number of IDRs (≥ 20 amino acid residues, predicted by at least three different predictors) with K/R-x(3,7)-K-x-K/R-K/R motif.

### RDPA proteins participate in the regulation of gene expression

**Fig 3:**
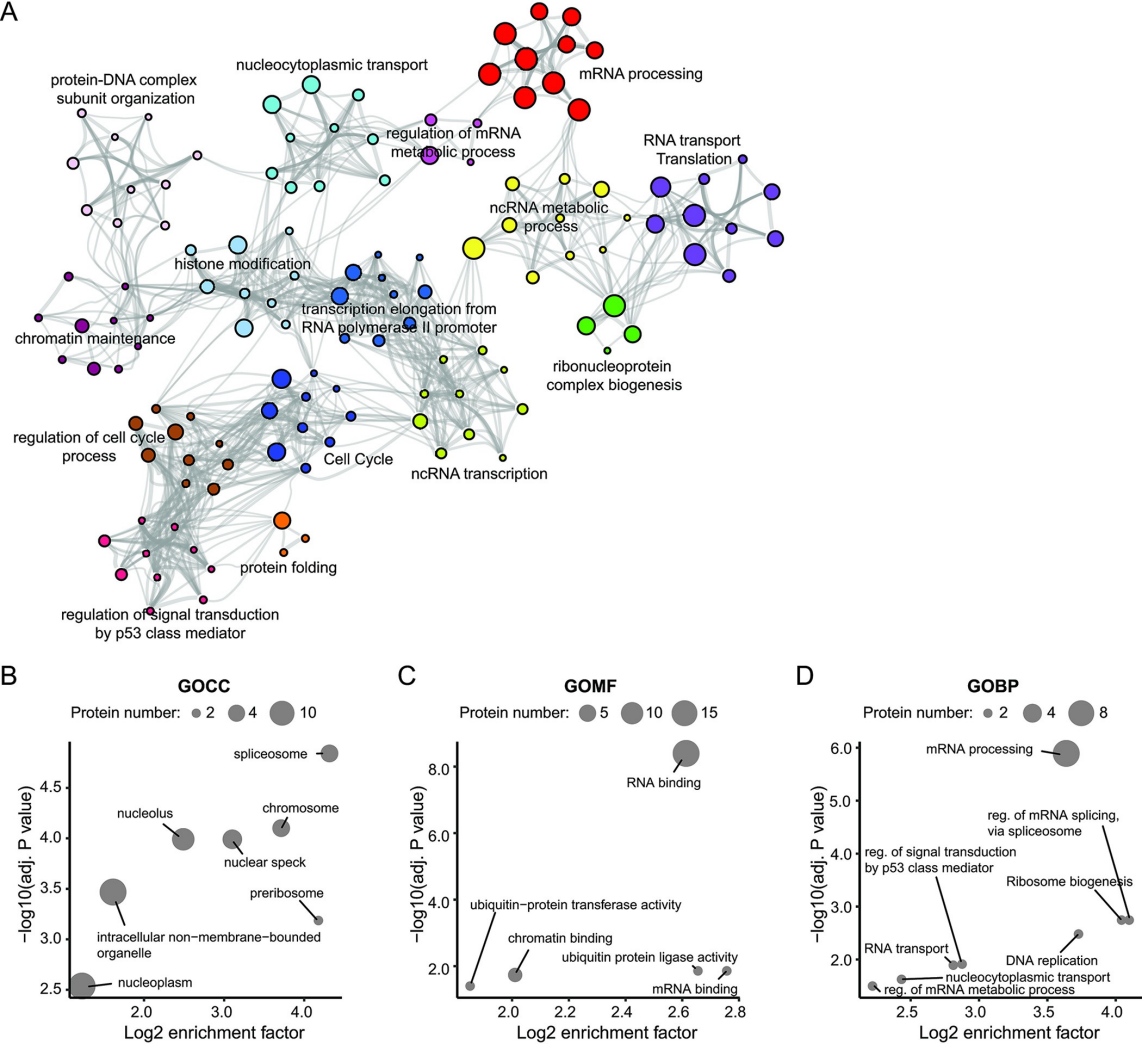
Functional analysis of the RDPA proteome. **A)** Hierarchical clustering of functional terms overrepresented in the RDPA proteome revealed enrichment of proteins regulating gene expression. Each node represents a term; the size and color represent the number of input genes and cluster identity, respectively. Terms with a similarity score > 0.3 are linked by an edge. Representative terms are selected for each cluster and shown as label. The analysis was performed in Metascape, with human proteome as a background. **B-D)** Gene ontology (GO) analysis of human proteins containing K/R-x(3,7)-K-x-K/R-K/R motif in IDRs using SLiMSearch tool based on **(B)** cellular compartment (GOCC), **(C)** molecular function (GOMF), and **(D)** biological process (GOBP). The y-axis shows the–log10 adjusted p-value (Fisher’s exact test) of proteins from a GO category, the x-axis shows the log2 enrichment factor. The size of the bubble corresponds to the number of proteins.

GO analysis provides valuable insights into the function of proteins identified by shotgun MS-based approaches. Therefore, the biological processes in which RDPA proteins are involved were analyzed using Metascape [71] compared to the human proteome. The results showed that RDPA proteins are mainly involved in different stages of gene expression, including chromatin accessibility, RNA transcription, RNA processing, and RNA transport (Fig 3A and S17 and S18 Tables). These processes, such as Pol2 transcription, RNA processing, or RNA export depend on the formation of distinct membraneless nuclear compartments in a process regulated by RNA [29,40]. This observation is consistent with our bioinformatic data (Fig 2) as well as with previously published data on PIP2 effectors [2,14,83,121]. Similar results were obtained using Nuclear fraction proteins as a background instead of human proteome. In addition, proteins associated with the response to dsRNA were identified in the RDPA proteome in this comparison (S19 and S20 Tables).

To verify the GO results, we took a reverse approach and screened the human proteome for the presence of PIP2-binding K/R motifs within the IDRs. We used a short and linear motif discovery tool—SLiMSearch [85]—with a stringent disorder score cut-off to have a high probability of the motifs being located within the IDRs. The SLiMSearch results showed that the K/R-x(3,7)-K-x-K/R-K/R motif was present in 29 proteins, the K/R-x(3,7)-K-x-K/R motif was present in 61 proteins and the K/R-x(3,7)-K-x-K motif was present in 43 proteins. We further investigated whether the K/R motifs are preferentially localized in nuclear IDR-containing proteins, as suggested by the data shown in Fig 2C. Indeed, GO localization analysis showed that all three K/R motifs are enriched in proteins associated with nuclear components such as nuclear speckles and nucleoli (Fig 3B). In addition, two shorter motifs were enriched for nuclear euchromatin and nucleosome (S16A and S17A Figs and S21, S24 and S27 Tables). Thus, these data are consistent with our previous observations and suggest that K/R motifs within the IDRs have specific roles in nuclear processes.

We further focused on elucidating the molecular functions of these proteins (Figs 3C, S16B and S17B and S22, S25 and S28 Tables). Our analysis confirmed that proteins with at least one of the three K/R motifs in the IDRs have RNA binding capacity. These data are consistent with our bioinformatic analysis, which found that RNA binding function is enriched in the RDPA proteome (Fig 2A). The RNA binding ability of nuclear proteins is an important feature for the formation of biomolecular condensates, supporting the notion that RNA is the key factor defining nuclear compartmentalization [90]. Next, we analyzed the biological processes of proteins with K/R motifs containing IDRs. Processes of RNA splicing, RNA transport, ribosome biogenesis, nucleocytoplasmic transport and regulation of signal transduction by p53 class mediator were enriched between all motifs (Fig 3D and S23, S26 and S29 Tables). In contrast, histone modifications, nucleosome positioning, transcription elongation from the RNA polymerase II promoter, and ncRNA metabolism were enriched specifically for proteins with shorter K/R motifs in IDRs (S16C and S17C Figs and S26 and S29 Tables).

Taken together, these results are consistent with the results of the RDPA proteome bioinformatics and GO analyses (Figs 2A, 2C, and 3A). Interestingly, SLiMSearch data suggest that proteins with K/R-x(3,7)-K-x-K/R-K/R motif in IDRs may have a different nuclear distribution and be involved in different processes than proteins with IDRs with shorter K/R motifs, indicating a degree of specificity for a particular site of action. We hypothesize that different K/R motifs may localize to different PIP2 nuclear subpopulations (nuclear speckles, nucleoli and NLIs) and thus PIP2 acts as a molecular wedge via RNA association to attract and retain different sets of RDPA proteins. The PIP2-dependent landscape of subnuclear localization has recently been suggested as an important determinant of nuclear architecture [96]. A similar dependence on K/R protein levels has been identified as an important determinant of protein phase separation and localization [33,122]. Furthermore, a recently published study shows that mutations in IDR-containing proteins are often associated with the formation of K/R frame shifts that alter their phase separation capacities, leading to cancer predispositions [34]. Thus, these data confirm that PIP2 is a key player affecting the nuclear localization of interacting proteins.

### RNA regulates the association of the RDPA protein BRD4 with PIP2

**Fig 4:**
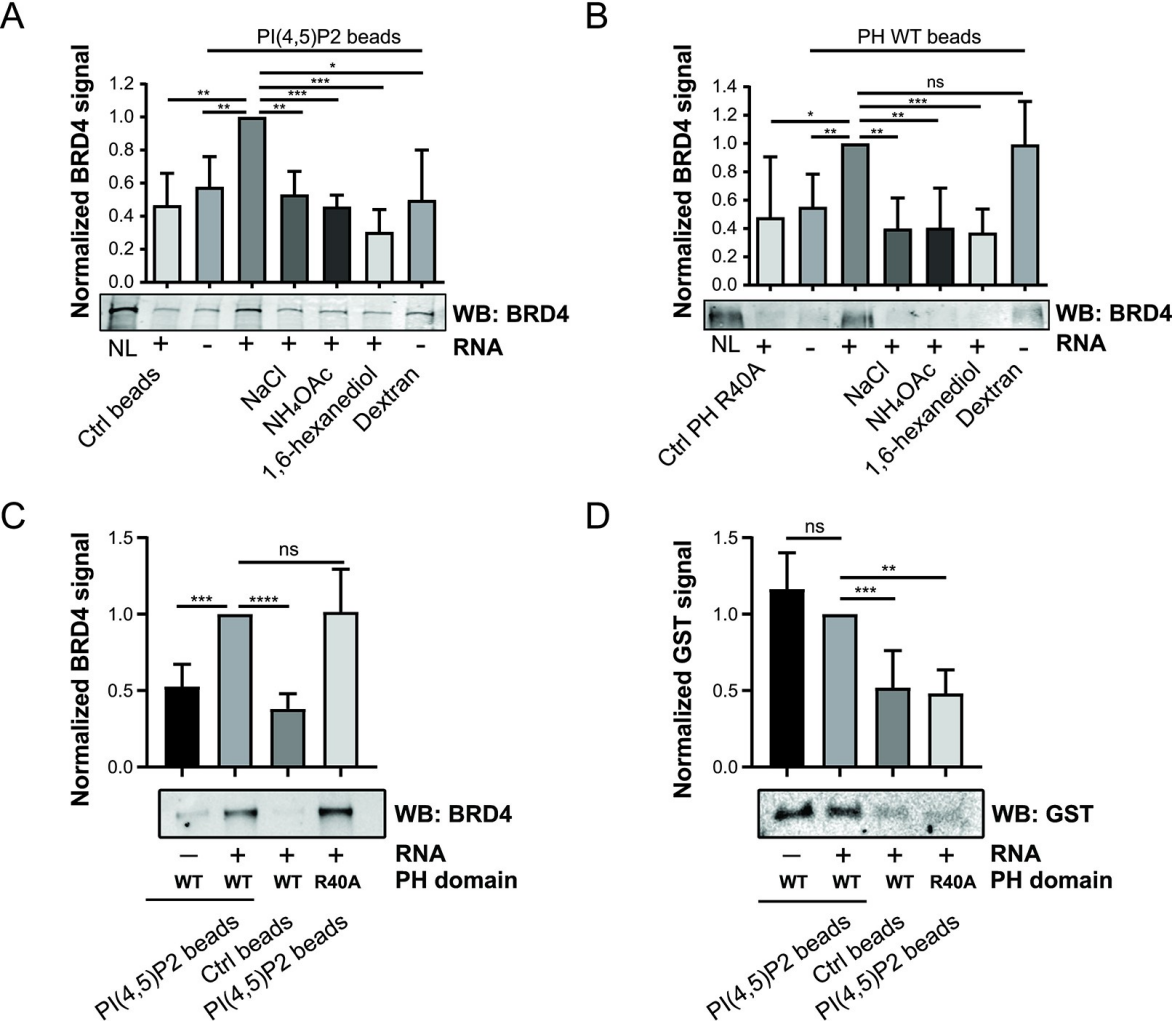
PIP2-conjugated agarose beads pull-down assays from nuclear lysates with added nuclear RNA extract upon different conditions. The PIP2 and empty control beads were incubated for 1 h at 4°C in nuclear lysates, washed, and subjected to WB detection of BDR4 protein **(A)**. WB signals at each pull-down condition in every repetition were normalized to the signal at PIP2 pull-down upon RNA addition condition. Statistical analysis was performed using Student’s t-test (n = 4). Error bars correspond to SEM. **(B)** GST-PLCδ1 PH pull-down assay testing the significance of PIP2 pull-down results for naturally occurring PIP2-BRD4 structures. WB signals at each pull-down condition in every repetition were normalized to the signal of GST-PLCδ1 PH WT domain pull-down upon RNA addition condition. Statistical analysis was performed using Student’s t-test (n = 6). Error bars correspond to SEM. The following treatments were used in the respective specimens as indicated in Fig 4A and 4B: the addition of 30 μg of nuclear RNA extract, 300 mM NaCl, 100 mM NH_4_OAc, 10% 1,6-hexanediol, and 10% dextran. **(C)** PIP2-conjugated beads pull-down assay with spike-in of recombinant GST-PLCδ1 PH domain testing the specificity of the effect of RNA on PIP2 binding of BRD4 protein. WB signals at each pull-down condition in every repetition were normalized to the signal from the PIP2-beads pull-down signal, wild-type GST-PLCδ1 PH domain, RNA addition condition. Statistical analysis was performed using Student’s t-test (n = 4). Error bars correspond to SEM. NL–nuclear lysate, PD–pull-down, * P < 0.05, ** P < 0.001, *** P < 0.0005.

Our bioinformatic analyses revealed that RDPA proteins contain charged K/R motifs within their IDRs, which are thought to be responsible for PIP2 recognition [2,83]. The RDPA protein Bromodomain-containing protein 4 (BRD4) is a transcriptional regulator with the ability to form phase-separated condensates *in vitro* and *in vivo* via its IDR [123]. To characterize the interactions between the selected RDPA protein BRD4 and PIP2, we performed pull-down experiments using PIP2-conjugated beads to mimic naturally occurring PIP2 structures under different conditions. Our results showed that protein BRD4 associates with PIP2 structures in the RNA-dependent manner, as the addition of exogenous RNA positively affected its PIP2 binding capacity (Fig 4A). Furthermore, the interactions between PIP2 and BRD4 protein are of electrostatic nature, as increased NaCl concentration (300 mM) significantly decreased PIP2 binding (Fig 4A). Biomolecular condensates are sensitive to increasing salt concentration because salt ions reduce weak electrostatic interactions necessary for cohesive forces within condensates [106,124]. NH4OAc treatment (100 mM) prevents the formation of structured RNA folds by denaturing RNA molecules, while leaving protein structures intact [40]. This treatment revealed that RNA-RNA interactions and the formation of higher-order folds (i.e., dsRNA) are important prerequisites for PIP2-BRD4 protein interactions (Fig 4A). The 1,6-hexanediol is often used to dissolve protein condensates formed by phase separation *in vitro* and *in vivo* [125], although the reliability of its use *in vivo* is still the subject of intense debate. Recent studies have shown that 1,6-hexanediol may affect the chromatin state and enzymatic functions of some proteins [126,127], making 1,6-hexanediol the drug of choice for *in vitro* assays only. The 1,6-hexanediol (10%) in the pull-down reaction reduced PIP2 association with the BRD4 protein (Fig 4A). The addition of the crowding agent dextran (10%) had no effect on the association of PIP2 with the BRD4 protein under conditions where no RNA was added (Fig 4A). In contrast, we observed an increased association of BRD4 with the PLC PH domain-conjugated beads in nuclear lysate pull-downs compared to the condition without RNA addition, suggesting that dextran may function similarly to RNA in enhancing BRD4 association with PIP2-containing complexes (Fig 4B). In addition, we performed the experiment with another RDPA protein, Cullin-associated and neddylation-dissociated protein 1 (CAND1). We observed an increased association of CAND1 with PIP2 beads induced by the addition of dextran (S18 Fig). CAND1 was previously identified as a protein associated with nuclear PIP2 during human papillomavirus infection [128]. Furthermore, the results of the pull-down through the GST-PLCδ1 PH domain of PIP2-containing nuclear material suggest the relevance of these observations to naturally occurring PIP2-BRD4 protein complexes (Fig 4B).

To further test the specific effect of RNA on BRD4 protein, we set up an experiment where we added the same amount of purified recombinant GST-tagged GST-PLCδ1 PH or its mutant variant R40A domains to the nuclear lysate as internal controls. We compared the effect of RNA extract addition on PIP2 association with BRD4 protein and the control PH domains. The addition of RNA extract increased the PIP2 association of BRD4 protein, whereas the association of the GST-PLCδ1 PH domain with PIP2 was decreased under these conditions (Fig 4C and 4D). These results suggest that the same amount of RNA that stimulates PIP2 association with K/R IDR-containing BRD4 proteins negatively affects PIP2 recognition by the canonical PIPs-binding PH domain. Thus, RNA appears to act as a local scaffold rather than a non-specific crowding agent. Furthermore, the R40A mutated PH domain did not bind to PIP2 beads in the presence of RNA and had no effect on BRD4-PIP2 association (Fig 4C). In addition, we provided evidence that the bivalent Mg^2+^ ions potentiate BRD4-PIP2 binding in the presence of RNA. Increasing the amount of this cation in the PIP2 bead pull-down sample led to a gradual increase in BRD4 binding, suggesting that this is an important prerequisite for the association (S19 Fig). Interestingly, high Mg^2+^ concentration (50 mM) led to a dramatic decrease in BRD4 binding, suggesting that there is a saturation point above which the ion molecules have a negative effect on binding. Based on these data, we hypothesize that the structured higher-order RNA binds PIP2 via Mg^2+^ and attracts the BRD4 protein, thus locally increasing its concentration and eventually leading to the formation of a condensate in the vicinity of a PIP2-containing surface. In addition, we tested the binding capacity of BRD4 to all existing PIPs in a pull-down experiment using PIP-conjugated agarose beads. Our results showed the highest association of BRD4 with PI(4,5)P2, and also small but significant binding to PI(3,4,5)P3 and to PI(5)P in the presence of RNA, suggesting a putative weak binding promiscuity (S20 Fig).

### BRD4 protein foci cluster in the vicinity to PIP2-containing nuclear structures

**Fig 5:**
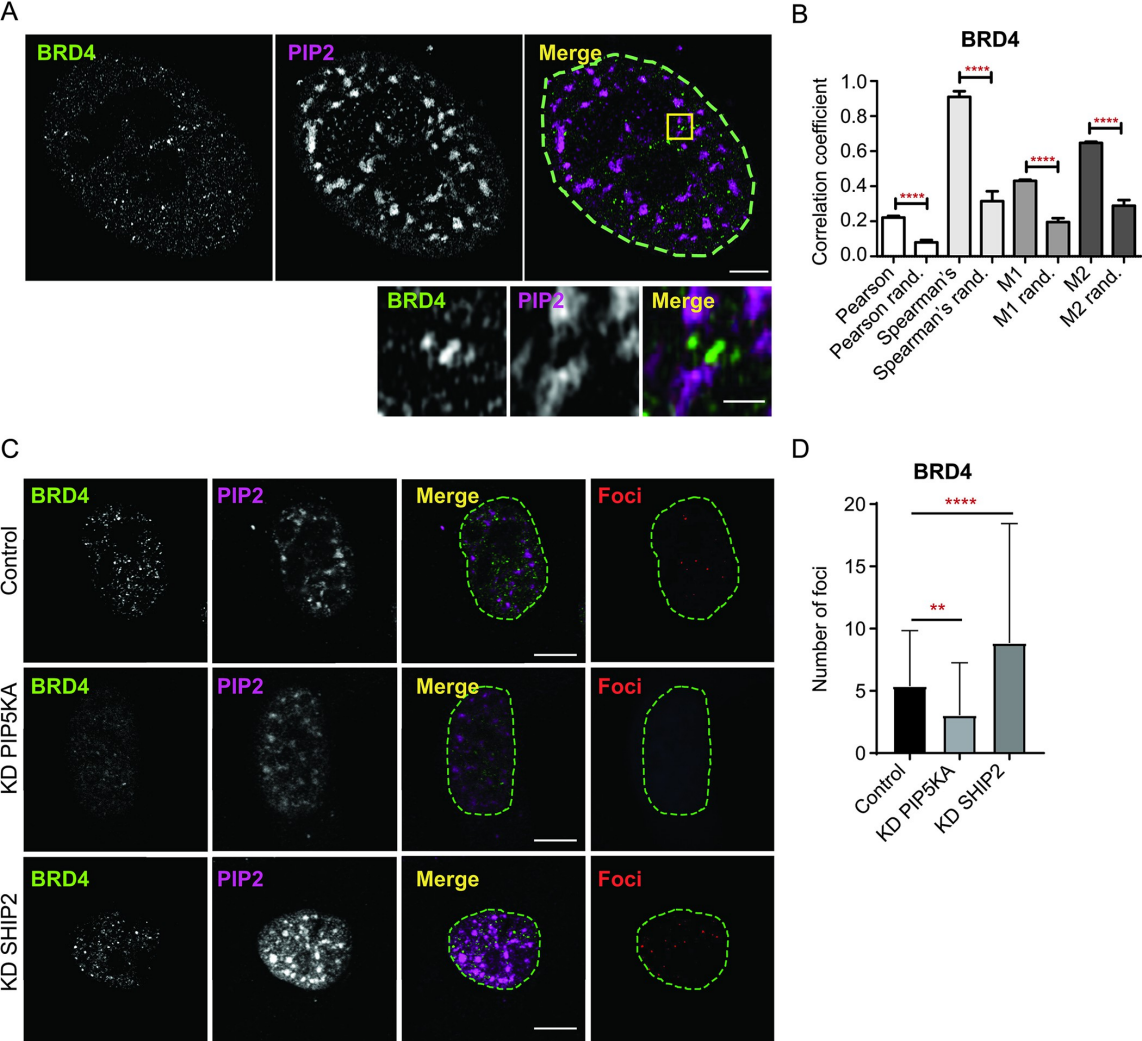
Localization of BRD4 in relation to PIP2 and changes in the number of BRD4 foci induced by the manipulation of PIP2 levels, visualized by super-resolution and confocal microscopy. **(A)** Representative images of immunofluorescence staining for BRD4 and PIP2 using specific antibodies show the localization of this protein in the vicinity of PIP2 in the nuclei of U2OS cells. Images were acquired by structured illumination microscopy (SIM). The inset shows a detail of BRD4 and PIP2 localization at a nucleoplasmic region within the U2OS cell nucleus (yellow square). Scale bars correspond to 5 μm and 1 μm resp. (inset). **B)** Statistical analysis of colocalization parameters by Pearson’s, Spearman’s, and Manders’ coefficients M1 and M2 compared to randomized images was performed using Student’s t-tests. Error bars correspond to SEM (** P < 0.005, *** P < 0.001, **** P < 0.0001), n = 3, N = 34 cells. **(C)** U2OS cells with decreased PIP2 levels by depletion of PIP5KA and increased PIP2 levels by depletion of SHIP2, respectively. Representative images show the localization of PIP2 and BRD4 using immunofluorescence staining. The last column shows the identified foci used for quantification of representative images in false red color, which does not represent the intensity of the signal. Scale bars correspond to 5 μm. **D)** The chart visualizes the average number of BRD4 foci identified per cell in knock-down (KD) control, KD PIP5KA, and KD SHIP2 U2OS cells. Statistical analysis was performed using Student’s t-tests (** P < 0.005; **** P < 0.0001), n = 4, N = 37 KD control cells, KD PIP5KA N = 44 cells, and KD SHIP2 N = 50 cells. Error bars correspond to SEM.

Nuclear PIP2 localizes to transcriptionally relevant compartments—nuclear speckles, NLIs, and nucleoli [11]. Therefore, we investigated the localization of the transcriptional regulator BRD4 in relation to nuclear PIP2 structures. To visualize the detailed localization of BRD4, we used super-resolution microscopy, which has recently been successfully applied to assess the co-patterning of nuclear proteins with PIP2 [96]. Our data showed that BRD4 formed foci in a dispersed pattern inside the nucleus (Fig 5A). We analyzed the colocalization of the signal of BRD4 protein with the PIP2 signal and compared the real data with the data randomized by rotating one channel with respect to the second channel [61]. Pearson’s, Spearman’s, and Manders’ coefficients were used to test signal colocalization and spatial co-distribution. Pearson’s coefficient measures the degree of correlative variation between the two channels while Spearman’s coefficient detects the mutual dependencies of two channel signals and thus measures the statistical association between these two channel signals. The Manders’ M1 and M2 coefficients measure the proportion of the intensity in each channel that coincides with an intensity in the other channel. The M1 and M2 coefficients are less dependent on the actual intensity ratios between the channels. Statistical analysis of the data revealed very limited colocalization of BRD4 protein with nuclear PIP2, but BRD4 rather clusters in the vicinity of PIP2 (Fig 5B).

### Number of BRD4 protein foci correlates with nuclear PIP2 levels

Based on the data presented so far, we propose that nuclear PIP2 attracts RDPA proteins containing K/R motifs, leading to their localization in the vicinity to PIP2-enriched areas. Therefore, the localization patterns of BRD4 protein should vary under conditions of different nuclear PIP2 levels. To test the effect of varying PIP2 levels on the localization of BRD4 protein, we experimentally increased nuclear PIP2 levels by depleting SHIP2 phosphatase and conversely decreased PIP2 levels by depleting PIP5KA (S21A Fig).

Image analysis and quantification of the microscopy data showed that increased PIP2 levels increased the number of BRD4 nuclear foci (Fig 5C and 5D). This effect may help to explain the importance of nuclear PIP2 in transcription and Pol2 condensation described elsewhere [11,14]. The total protein level of BRD4 was slightly lowered upon SHIP2 depletion, suggesting that increase in BRD4 protein foci number is not due to increased total protein concentration, but rather due to changes in its local level (S21B Fig). To exclude the possibility of an indirect effect of siRNA-mediated depletion on BRD4, we used SHIP2 inhibitors (K149), which increased nuclear PIP2 levels (S22 Fig). These data are consistent with siRNA depleted SHIP2 since BRD4 foci number were increased upon SHIP2 inhibition (S22 Fig) and suggest that the changes in BRD4 foci are indeed in correlation to nuclear PIP2 levels and not an indirect result of depletion of the SHIP2 enzyme. In addition, we tested the role of RNA in the formation of BRD4 foci. We recapitulated our experimental setup where RNase III was used to treat semi-permeabilized cells and measured the number of BRD4 condensates within the nucleus. These results showed that the number of BRD4 foci decreased upon removal of higher order RNA (S23 Fig). These data are consistent with the previously observed effect of RNA on the condensation capacity of BRD4 *in vitro* [40] and suggest that both higher-order RNA and nuclear PIP2 must be in fine balance to ensure that the correct number of BRD4 foci is formed.

Altogether our data indicate that the levels of nuclear PIP2 and higher-order RNA do indeed influence the formation or stability of BRD4 protein foci, presumably by altering their local concentration. The presence of an amphiphilic molecule in the nuclear environment has been proposed to explain the formation of so-called microemulsions, which are typically organized in nuclear processes [129]. Based on our data, we propose that nuclear PIP2 regulates the affinity between RNA and BRD4 protein. This interaction defines the areas of foci formation, thus orchestrating nuclear subcompartmentalization and leading to efficient gene expression. Our data are consistent with the recently published work in which the authors describe, among other fundamental findings, the effect of PIPs on the quantity, size and morphology of condensates in an *in vitro* system [59]. This model brings new perspectives to another recent observation that carcinogenic human papillomavirus (HPV) infection increases nuclear PIP2 levels in human wart samples [97]. Thus, we provide mechanistic insights into the real biological implications of this phenomenon.

In conclusion, we have shown that nuclear PIP2 localization is dependent on the presence of higher-order RNA and identified RDPA proteins that associate with nuclear PIP2 in a higher-order RNA-dependent manner. To this end, we developed and optimized a MS-based experimental pipeline that can be applied to other nuclear lipid-protein interactors. Our results showed that interactions between RDPA protein BRD4 and PIP2 are mediated by electrostatic interactions, presumably via the enriched K/R motifs within the IDRs. The PIP2-binding function of such K/R motifs has been described previously [3,83]. Based on our bioinformatics analysis, we have shown that more than half of the RDPA proteins have experimentally demonstrated RNA binding capacity and are associated with the process of phase separation. We found that IDRs have a bimodal distribution of pI, and we provide bioinformatic tools for their convenient analyses. We also showed that IDRs of RDPA proteins are longer and more hydrophilic. The K/R motif-containing IDRs of RDPA proteins are enriched for known phosphorylation, acetylation, and ubiquitination sites, possibly providing an additional level of regulation for their integration into phase-separated condensates. GO analysis revealed that RDPA proteins are involved in RNA transcription, RNA processing and transport, translation, cell cycle regulation, histone modification, and chromatin maintenance. The involvement of proteins containing IDRs with K/R motifs in similar processes was confirmed by our SLIMsearch analysis. We showed the localization of RDPA protein BRD4 with respect to nuclear PIP2 structures. Finally, we have shown that increased levels of nuclear PIP2 lead to an increased number of BRD4 foci and conversely, the removal of higher-order RNA leads to a decreased number of BRD4 foci.

## Supporting information

S1 Fig The effect of RNase III treatment on PIP2 and SON localization in non-permeabilized cell nuclei visualized by immunofluorescence staining. **(A)** U2OS cells were treated by RNase III without a semi-permeabilization step, and subsequently stained with PIP2 and SON-specific antibodies. Images were acquired by fluorescence microscopy. **B)** Quantification of normalized mean PIP2 signal intensity levels after RNase III treatment in segmented nuclei for nuclear speckles (Sp) and nucleoplasm (Np) regions without semi-permeabilization step (orange bars) compared to mock and RNase III-treated semi-permeabilized U2OS cells (Fig 1C). Scale bars correspond to 5 μm. Statistical analysis was performed using Student’s t-tests. Error bars correspond to SEM (**** P < 0.0001), n = 3, N = 76 mock-treated cells, N = 89 RNase III-treated semi-permeabilized cells, N = 58 RNase III-treated non-permeabilized cells.
(PDF)

S2 Fig Localization of PIP2 signals visualized by the combination of specific antibody and GST-tagged GST-PLCδ1 PH domain in U2OS cell nucleus. **(A)** Representative images of immunofluorescence staining for PIP2 using specific antibody and anti-GST antibody to visualize the PLCδ1 PH domain signal show the colocalization of PIP2 in the nuclei of U2OS cells. Images were captured by confocal microscopy. Scale bars correspond to 5 μm. **B)** Statistical analysis of colocalization parameters by Pearson’s, Spearman’s and Manders’ coefficients M1 and M2 compared to random images was performed using Student’s t-tests. Error bars correspond to SEM (**** P < 0.0001), n = 3, N = 34 cells.
(PDF)

S3 Fig Overview and overlaps between the datasets as identified by mass spectrometry analyses (A-C). Numbers represent proteins from Majority protein IDs mapped to UniProt (release 2022_01). Datasets Nucleo-specific proteins **(A)**, Nuclear fraction proteins **(B)** and Total cell proteome **(C)** were supplemented by missing RDPA proteins (for more information see S3 and S4 Tables). The modified datasets were then used for the bioinformatic analyses presented in this study (related to Fig 2).
(PDF)

S4 Fig Additional bioinformatic analyses of RDPA proteome features (related to Fig 2A and 2B). **A)** RDPA proteome is significantly enriched for RNA-binding, phase separation capacity, and combination of both properties. **B-C)** RDPA proteome is enriched for IDRs longer than 30 amino acid residues predicted by ESpritz X-Ray (X-Ray), ESpritz Disprot (Disprot), and ESpritz NMR (NMR). Statistical analysis was performed using a hypergeometric test (ns not significant, * P < 0.05, ** P < 0.01, and *** P < 0.001).
(PDF)

S5 Fig Additional bioinformatic analyses of RDPA proteome features (related to Fig 2C). **A-B)** Enrichment of K/R motifs in the RDPA proteome. These motifs were abundantly present in the RDPA proteome, but only the K/R-x(3,7)-K-x-K/R-K/R motif (the longest one) was significantly enriched, compared to all other datasets. **C)** Percentage of PIP2-binding K/R motif sites localized in IDRs (from all K/R motif sites in the dataset) is elevated in RDPA proteome (only IDRs predicted by at least three different predictors with minimal length 20 amino acid residues were considered). Statistical analysis was performed using a hypergeometric test (* P < 0.05, ** P < 0.01, and *** P < 0.001).
(PDF)

S6 Fig Additional bioinformatic analysis of RDPA proteome features (relevant to Fig 2D). Bimodal pI distribution of IDRs predicted by nine different IDR predictors (Database of Disordered Protein Predictions - https://d2p2.pro/). Only IDRs with minimal length of 20 amino acid residues were considered.
(PDF)

S7 Fig Additional bioinformatic analyses of RDPA proteome features (relevant to Fig 2D–2F). **A)** pI values of IDRs predicted by ESpritz X-Ray in the analyzed protein datasets show a bimodal distribution. **B)** The IDRs in the “acidic” population (pI < 7) are enriched with D/E amino acid residues. **C)** The IDRs in the “basic” population (pI > 7) are K/R-rich.
(PDF)

S8 Fig Additional bioinformatic analyses of RDPA proteome features (relevant to Fig 2E and 2F). **A-B)** Distribution of the numbers of acidic **(A)** and basic **(B)** residues in IDRs predicted by nine different IDR predictors (Database of Disordered Protein Predictions; only IDRs with minimal length of 20 amino acid residues were considered) in the “main” datasets. **C-D)** Distribution of the numbers of acidic **(C)** and basic **(D)** residues in IDRs predicted by nine different IDR predictors (Database of Disordered Protein Predictions; only IDRs with minimal length of 20 amino acid residues were considered) in the “additional” datasets.
(PDF)

S9 Fig Additional bioinformatic analysis of RDPA proteome features (relevant to Fig 2D and 2G). **A-C)** Distribution of the log2 transformed length of all IDRs **(A)** or IDRs that were acidic (pI < 7) **(B)** or basic (pI > 7) **(C)** and predicted by nine different IDR predictors (Database of Disordered Protein Predictions; only IDRs with minimal length of 20 amino acid residues were considered) in the “main” datasets.
(PDF)

S10 Fig Additional bioinformatic analysis of RDPA proteome features (relevant to Fig 2D and 2G). **A-C)** Distribution of the log2 transformed length of all IDRs **(A)** or IDRs that were acidic (pI < 7) **(B)** or basic (pI > 7) **(C)** and predicted by nine different IDR predictors (Database of Disordered Protein Predictions; only IDRs with minimal length of 20 amino acid residues were considered) in the “additional” datasets.
(PDF)

S11 Fig Additional bioinformatic analysis of RDPA proteome features (relevant to Fig 2G). **A-C)** RDPA proteins IDRs containing the three K/R motifs tend to be significantly longer compared to the other six datasets. **A)** Boxplots show the distributions of log10 transformed length. **B)** Density plots of the IDR lengths highlight the presence of two populations of longer IDRs in the RDPA proteome and Nucleo-specific proteins (red arrows). **C)** The P values of all pairwise comparisons between the datasets and motifs were estimated by a pairwise Wilcox test. Benjamini-Hochberg correction was applied to correct for multiple hypothesis testing. Ref.–reference, prot.–proteome, fr.–fraction, spec.–specific.
(PDF)

S12 Fig Additional bioinformatic analysis of RDPA proteome features (relevant to Fig 2B and 2G). The percentage of disordered amino acid residues in IDRs predicted by ESpritz X-Ray (X-Ray), ESpritz Disprot (Disprot), and ESpritz NMR (NMR) in the “main” **(A)** and “additional” **(B)** datasets. Statistical analysis was performed using a hypergeometric test (*** P < 0.001).
(PDF)

S13 Fig Additional bioinformatic analysis of RDPA proteome features (relevant to Fig 2H). **A)** Boxplots show the distributions of pI values of IDRs between datasets and K/R motifs. **B)** Density plots of the IDR pI values highlight the presence of bimodal distributions. **C)** The P values of all pairwise comparisons between the datasets and motifs were estimated by a pairwise Wilcox test. Benjamini-Hochberg correction was applied to correct for multiple hypothesis testing. Ref.–reference, prot.–proteome, fr.–fraction, spec.–specific.
(PDF)

S14 Fig Additional bioinformatic analysis of RDPA proteome features (relevant to Fig 2I). **A-B)** Distribution of the GRAVY scores of IDRs predicted by nine different IDR predictors (Database of Disordered Protein Predictions; only IDRs with minimal length of 20 amino acid residues were considered) in the “main” **(A)** and “additional” **(B)** datasets.
(PDF)

S15 Fig Additional bioinformatic analysis of RDPA proteome features (relevant to Fig 2I). **A)** Boxplots show the distributions of the GRAVY score of IDRs between datasets and K/R motifs. **B)** Density plots of the IDR GRAVY scores. **C)** The P values of all pairwise comparisons between the datasets and motifs were estimated by a pairwise Wilcox test. Benjamini-Hochberg correction was applied to correct for multiple hypothesis testing. Ref.–reference, prot.–proteome, fr.–fraction, spec.–specific.
(PDF)

S16 Fig Functional analysis of the RDPA proteome (relevant to Fig 4B–4D). **A-C)** Gene ontology (GO) analysis of human proteins containing K/R-x(3,7)-K-x-K/R motif in IDRs using SLiMSearch tool based on **(A)** cellular compartment (GOCC), **(B)** molecular function (GOMF), and **(C)** biological process (GOBP). The y-axis shows the -log10 adjusted p-value (Fisher’s exact test) of proteins from a GO category, the x-axis shows the log2 enrichment factor. The size of the bubble corresponds to the number of proteins.
(PDF)

S17 Fig Functional analysis of the RDPA proteome (relevant to Fig 4B–4D). **A-C)** Gene ontology (GO) analysis of human proteins containing K/R-x(3,7)-K-x-K motif in IDRs using SLiMSearch tool based on **(A)** cellular compartment (GOCC), **(B)** molecular function (GOMF), and **(C)** biological process (GOBP). The y-axis shows the -log10 adjusted p-value (Fisher’s exact test) of proteins from a GO category, the x-axis shows the log2 enrichment factor. The size of the bubble corresponds to the number of proteins.
(PDF)

S18 Fig PIP2-conjugated agarose beads pull-down assays from nuclear lysates with added nuclear RNA extract upon different conditions. The PIP2 and empty control beads were incubated for 1 h at 4°C in nuclear lysates, washed, and subjected to WB detection of CAND1 protein. WB signals at each pull-down condition in every repetition were normalized to the signal at PIP2 pull-down upon RNA addition condition. Statistical analysis was performed using Student’s t-test (n = 3). Error bars correspond to SEM (NL–nuclear lysate, * P < 0.05, ** P < 0.001, *** P < 0.0005, **** P < 0.0001). The following treatments were used in the respective specimens as indicated in Fig 4A and 4B: the addition of 30 μg of nuclear RNA extract, 300 mM NaCl, 100 mM NH_4_OAc, 10% 1,6-hexanediol, and 10% dextran.
(PDF)

S19 Fig PIP2-conjugated agarose beads pull-down assays from nuclear lysates with the addition of 30 μg nuclear RNA extract at increasing concentrations of Mg^2+^. PIP2 beads were incubated in nuclear lysates for 1 h at 4°C, washed, and subjected to WB detection of BRD4 protein. WB signals for each pull-down condition in each replicate were normalized to the highest signal (25 mM Mg^2+^). Statistical analysis was performed by Student’s t-test (n = 3). Error bars correspond to SEM.
(PDF)

S20 Fig Different PIPs-conjugated agarose beads pull-down assays from nuclear lysates with the addition of 30 μg nuclear RNA extract. PIPs and control empty beads were incubated in nuclear lysates for 1 h at 4°C, washed, and subjected to WB detection of BRD4 protein. WB signals for each pull-down condition in each replicate were normalized to the highest signal (PI(4,5)P2). Statistical analysis was performed by Student’s t-test (n = 4). Error bars correspond to SEM (** P < 0.001, **** P < 0.0001).
(PDF)

S21 Fig Manipulation of PIP2 level by PIP5KA and SHIP2 knock-down (relevant to Fig 5C and 5D). **(A)** Microscopy confirmation of the manipulation of PIP2 levels induced by depletion of PIP5KA and SHIP2 enzymes. Statistical analysis was performed using Student’s t-tests (**** P < 0.0001), n = 3, n = 4, N = 37 KD control cells, KD PIP5KA N = 44, and KD SHIP2 N = 50 cells, respectively). Error bars correspond to SEM. **(B)** WB analysis of the efficacy of PIP5KA and SHIP2 siRNA depletion and its effect on BRD4 protein levels.
(PDF)

S22 Fig Confocal microscopy visualization of changes in the number of BRD4 foci induced by SHIP2 inhibition. **(A)** Representative figures show the localization of PIP2 and BRD4 using immunofluorescence staining. The last column shows the identified foci in false red color, which does not represent the intensity of the signal. Scale bars correspond to 5 μm. **B)** Quantification of PIP2 levels upon K149 treatment in U2OS cells. **(C)** The chart visualizes the average number of BRD4 foci identified per cell in control and SHIP2 inhibited U2OS cells. Statistical analysis was performed using Student’s t-tests (**** P < 0.0001), n = 5, N = 56 control cells, N = 62 SHIP2 inhibited cells). Error bars correspond to SEM.
(PDF)

S23 Fig Changes in the number of BRD4 foci induced by the RNase III treatment in semi-permeabilized U2OS cells visualized by confocal microscopy. **(A)** Representative figures show the localization of PIP2 and BRD4 using immunofluorescence staining. The last column shows the identified foci in false red color, which does not represent the intensity of the signal. Scale bars correspond to 5 μm. **B)** The chart visualizes the average number of BRD4 foci identified per cell in non-treated and RNase III treated semi-permeabilized U2OS cells. Statistical analysis was performed using Student’s t-tests (**** P < 0.0001), n = 4, N = 46 non-treated cells, N = 64 RNase III treated cells). Error bars correspond to SEM.
(PDF)

S1 Table Quantifiable proteins from mass spectrometry analysis of RNA-dependent PIP2-associated (RDPA) nuclear proteome. (XLSX)

S2 Table Quantifiable proteins from mass spectrometry analysis of nuclear fraction and cytosolic fraction proteomes. (XLSX)

S3 Table Protein datasets employed for bioinformatics. (XLSX)

S4 Table UniProtKB accession numbers for protein datasets employed for bioinformatics. (XLSX)

S5 Table Proteins with PIP2-binding domain(s) in analysed datasets. (XLSX)

S6 Table RNA-binding proteins in analysed datasets. (XLSX)

S7 Table Proteins connected with phase separation or membraneless organelles in analysed datasets. (XLSX)

S8 Table RNA-binding proteins connected with phase separation or membraneless organelles in analysed datasets. (XLSX)

S9 Table Prediction of intrinsically disordered regions in proteins of analysed datasets. (XLSX)

S10 Table Presence of K/R motifs in proteins of analysed datasets. (XLSX)

S11 Table Presence of K/R motifs in intrinsically disordered regions of proteins of analysed datasets. (XLSX)

S12 Table Analysis of lengths of intrinsically disordered regions containing K/R motifs. (XLSX)

S13 Table Analysis of specific enrichment of longer intrinsically disordered regions containing K/R motifs between datasets. (XLSX)

S14 Table Analysis of isoelectric points of intrinsically disordered regions containing K/R motifs. (XLSX)

S15 Table Analysis of hydrophobicity of intrinsically disordered regions containing K/R motifs. (XLSX)

S16 Table Presence of posttranslational modification sites within intrinsically disordered regions containing K/R motifs. (XLSX)

S17 Table Annotated data of RNA-dependent PIP2-associated (RDPA) proteome for gene ontology analysis against human proteome by Metascape. (XLSX)

S18 Table Gene ontology analysis of biological processes associated with RNA-dependent PIP2-associated (RDPA) proteome compared to human proteome. (XLSX)

S19 Table Annotated data of RNA-dependent PIP2-associated (RDPA) proteome for gene ontology analysis against Nuclear fraction proteins by Metascape. (XLSX)

S20 Table Gene ontology analysis of biological processes associated with RNA-dependent PIP2-associated (RDPA) proteome compared to Nuclear fraction proteins. (XLSX)

S21 Table Gene ontology analysis of cellular localization of proteins containing motif K/R-x(3,7)-K-x-K/R-K/R in their intrinsically disordered regions. (XLSX)

S22 Table Gene ontology analysis of molecular functions of proteins containing motif K/R-x(3,7)-K-x-K/R-K/R in their intrinsically disordered regions. (XLSX)

S23 Table Gene ontology analysis of biological processes associated with proteins containing motif K/R-x(3,7)-K-x-K/R-K/R in their intrinsically disordered regions. (XLSX)

S24 Table Gene ontology analysis of cellular localization of proteins containing motif K/R-x(3,7)-K-x-K/R in their intrinsically disordered regions. (XLSX)

S25 Table Gene ontology analysis of molecular functions of proteins containing motif K/R-x(3,7)-K-x-K/R in their intrinsically disordered regions. (XLSX)

S26 Table Gene ontology analysis of biological processes associated with proteins containing motif K/R-x(3,7)-K-x-K/R in their intrinsically disordered regions. (XLSX)

S27 Table Gene ontology analysis of cellular localization of proteins containing motif K/R-x(3,7)-K-x-K in their intrinsically disordered regions. (XLSX)

S28 Table Gene ontology analysis of molecular functions of proteins containing motif K/R-x(3,7)-K-x-K in their intrinsically disordered regions. (XLSX)

S29 Table Gene ontology analysis of biological processes associated with proteins containing motif K/R-x(3,7)-K-x-K in their intrinsically disordered regions. (XLSX)
