# Supplementary material for: The RNA-dependent association of phosphatidylinositol 4,5-bisphosphate with intrinsically disordered proteins contribute to nuclear compartmentalization
Source: PLoS Genet. 2024 Dec 2;20(12):e1011462. doi: 10.1371/journal.pgen.1011462 (PMC11668513; doi:10.1371/journal.pgen.1011462)
Supplement: S1 Fig — (A) U2OS cells were treated by RNase III without a semi-permeabilization step, and subsequently stained with PIP2 and SON-specific antibodies. Images were acquired by fluorescence microscopy. B) Quantification of normalized mean PIP2 signal intensity levels after RNase III treatment in segmented nuclei for nuclear speckles (Sp) and nucleoplasm (Np) regions without semi-permeabilization step (orange bars) compared to mock and RNase III-treated semi-permeabilized U2OS cells (Fig 1C). Scale bars correspond to 5 μm. Statistical analysis was performed using Student’s t-tests. Error bars correspond to SEM (**** P < 0.0001), n = 3, N = 76 mock-treated cells, N = 89 RNase III-treated semi-permeabilized cells, N = 58 RNase III-treated non-permeabilized cells. (PDF) [file pgen.1011462.s001.pdf]

S1 Fig

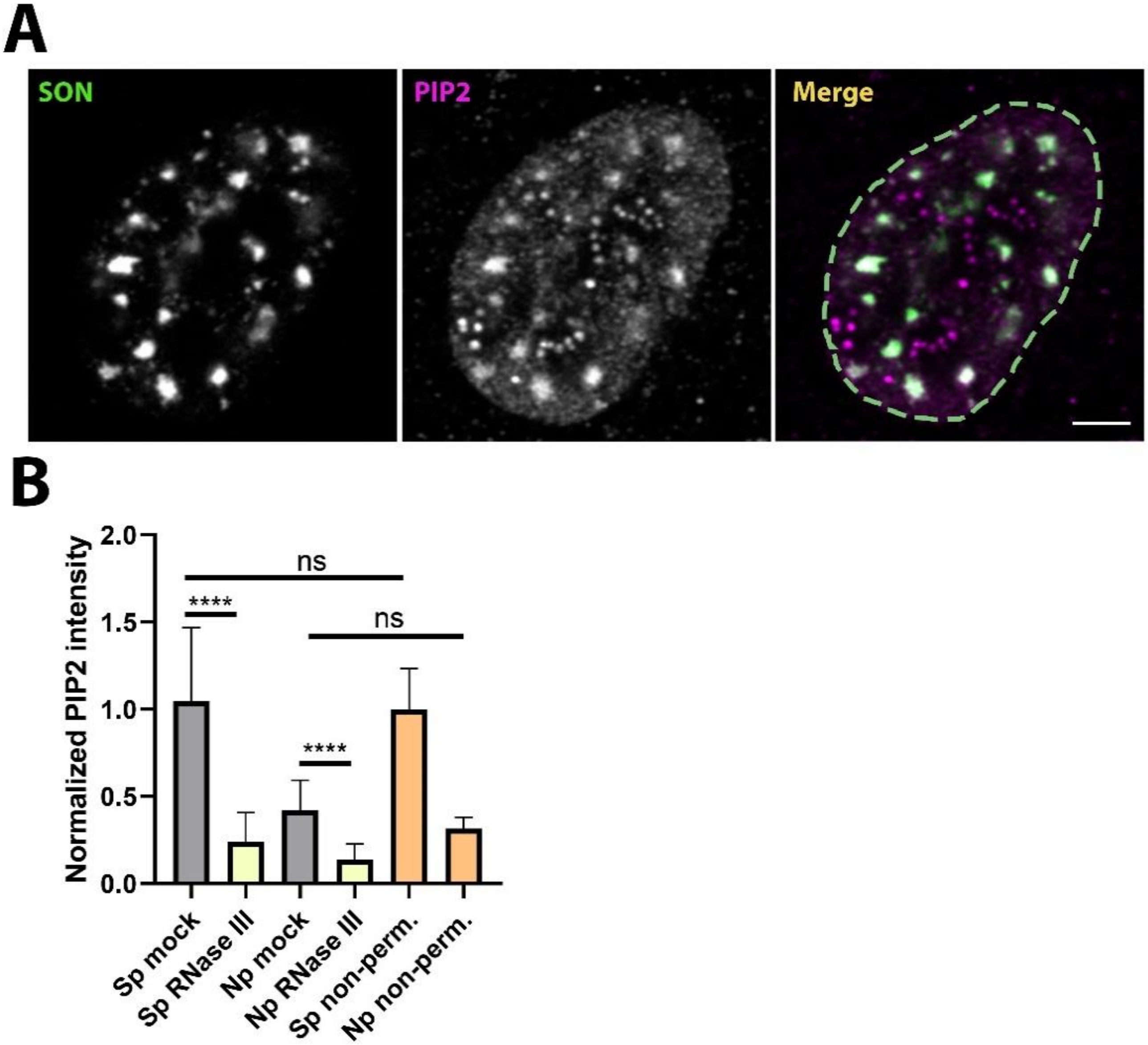

S1 Fig. The effect of RNase III treatment on PIP2 and SON localization in non-permeabilized cell nuclei visualized by immunofluorescence staining. (A) U2OS cells were treated by RNase III without a semi-permeabilization step, and subsequently stained with PIP2 and SON-specific antibodies. Images were acquired by fluorescence microscopy. **B)** Quantification of normalized mean PIP2 signal intensity levels after RNase III treatment in segmented nuclei for nuclear speckles (Sp) and nucleoplasm (Np) regions without semi-permeabilization step (orange bars) compared to mock and RNase III-treated semi-permeabilized U2OS cells (Fig 1C). Scale bars correspond to 5 µm. Statistical analysis was performed using Student's t-tests. Error bars correspond to SEM (**** $P < 0.0001$), $n = 3$, $N = 76$ mock-treated cells, $N = 89$ RNase III-treated semi-permeabilized cells, $N = 58$ RNase III-treated non-permeabilized cells.
