# Supplementary material for: The RNA-dependent association of phosphatidylinositol 4,5-bisphosphate with intrinsically disordered proteins contribute to nuclear compartmentalization
Source: PLoS Genet. 2024 Dec 2;20(12):e1011462. doi: 10.1371/journal.pgen.1011462 (PMC11668513; doi:10.1371/journal.pgen.1011462)
Supplement: S2 Fig — (A) Representative images of immunofluorescence staining for PIP2 using specific antibody and anti-GST antibody to visualize the PLCδ1 PH domain signal show the colocalization of PIP2 in the nuclei of U2OS cells. Images were captured by confocal microscopy. Scale bars correspond to 5 μm. B) Statistical analysis of colocalization parameters by Pearson’s, Spearman’s and Manders’ coefficients M1 and M2 compared to random images was performed using Student’s t-tests. Error bars correspond to SEM (**** P < 0.0001), n = 3, N = 34 cells. (PDF) [file pgen.1011462.s002.pdf]

S2 Fig

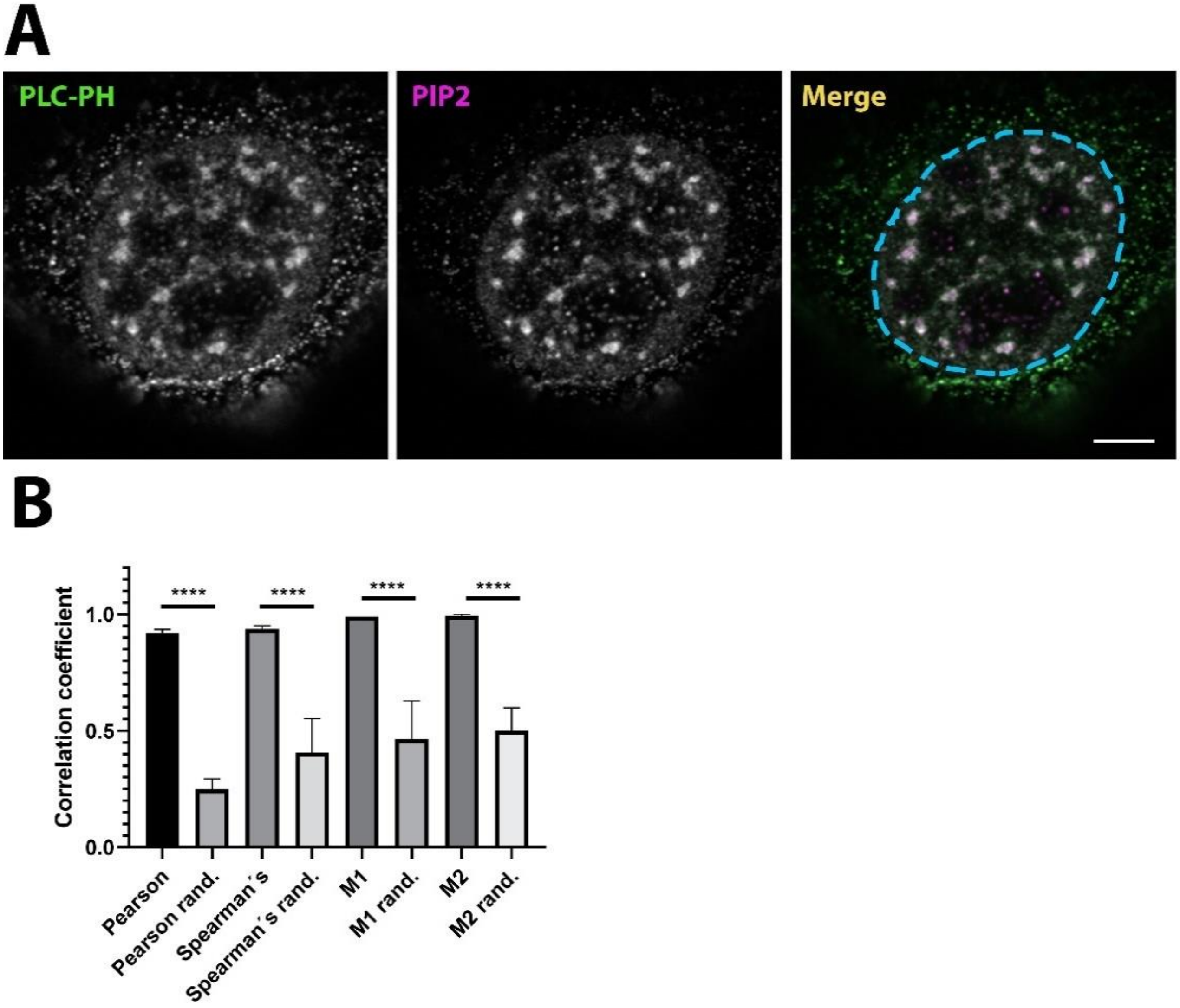

S2 Fig. Localization of PIP2 signals visualized by the combination of specific antibody and GST-tagged GST-PLCδ1 PH domain in U2OS cell nucleus. (A) Representative images of immunofluorescence staining for PIP2 using specific antibody and anti-GST antibody to visualize the PLCδ1 PH domain signal show the colocalization of PIP2 in the nuclei of U2OS cells. Images were captured by confocal microscopy. Scale bars correspond to 5 µm. **B)** Statistical analysis of colocalization parameters by Pearson's, Spearman's and Manders' coefficients M1 and M2 compared to random images was performed using Student's t-tests. Error bars correspond to SEM (**** $P < 0.0001$), $n = 3$, $N = 34$ cells.
