# Supplementary material for: The RNA-dependent association of phosphatidylinositol 4,5-bisphosphate with intrinsically disordered proteins contribute to nuclear compartmentalization
Source: PLoS Genet. 2024 Dec 2;20(12):e1011462. doi: 10.1371/journal.pgen.1011462 (PMC11668513; doi:10.1371/journal.pgen.1011462)
Supplement: S3 Fig — Overview and overlaps between the datasets as identified by mass spectrometry analyses (A-C). Numbers represent proteins from Majority protein IDs mapped to UniProt (release 2022_01). Datasets Nucleo-specific proteins (A), Nuclear fraction proteins (B) and Total cell proteome (C) were supplemented by missing RDPA proteins (for more information see S3 and S4 Tables). The modified datasets were then used for the bioinformatic analyses presented in this study (related to Fig 2). (PDF) [file pgen.1011462.s003.pdf]

S3 Fig

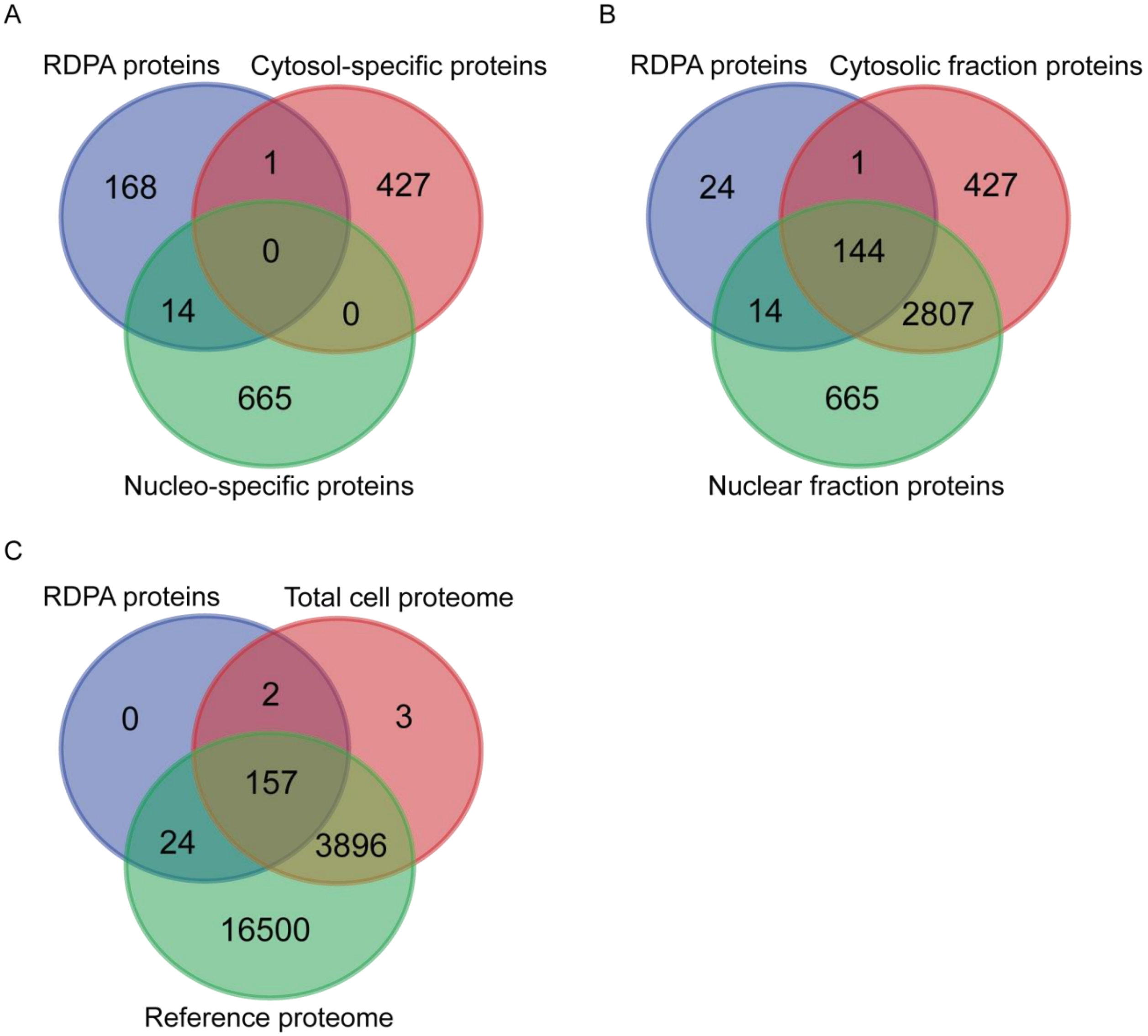

S3 Fig. Overview and overlaps between the datasets as identified by mass spectrometry analyses (A-C). Numbers represent proteins from Majority protein IDs mapped to UniProt (release 2022_01). Datasets Nucleo-specific proteins **(A)**, Nuclear fraction proteins **(B)** and Total cell proteome **(C)** were supplemented by missing RDPA proteins (for more information see S3 and S4 Tables). The modified datasets were then used for the bioinformatic analyses presented in this study (related to Fig 2).
