# Supplementary material for: The RNA-dependent association of phosphatidylinositol 4,5-bisphosphate with intrinsically disordered proteins contribute to nuclear compartmentalization
Source: PLoS Genet. 2024 Dec 2;20(12):e1011462. doi: 10.1371/journal.pgen.1011462 (PMC11668513; doi:10.1371/journal.pgen.1011462)
Supplement: S4 Fig — A) RDPA proteome is significantly enriched for RNA-binding, phase separation capacity, and combination of both properties. B-C) RDPA proteome is enriched for IDRs longer than 30 amino acid residues predicted by ESpritz X-Ray (X-Ray), ESpritz Disprot (Disprot), and ESpritz NMR (NMR). Statistical analysis was performed using a hypergeometric test (ns not significant, * P < 0.05, ** P < 0.01, and *** P < 0.001). (PDF) [file pgen.1011462.s004.pdf]

S4 Fig

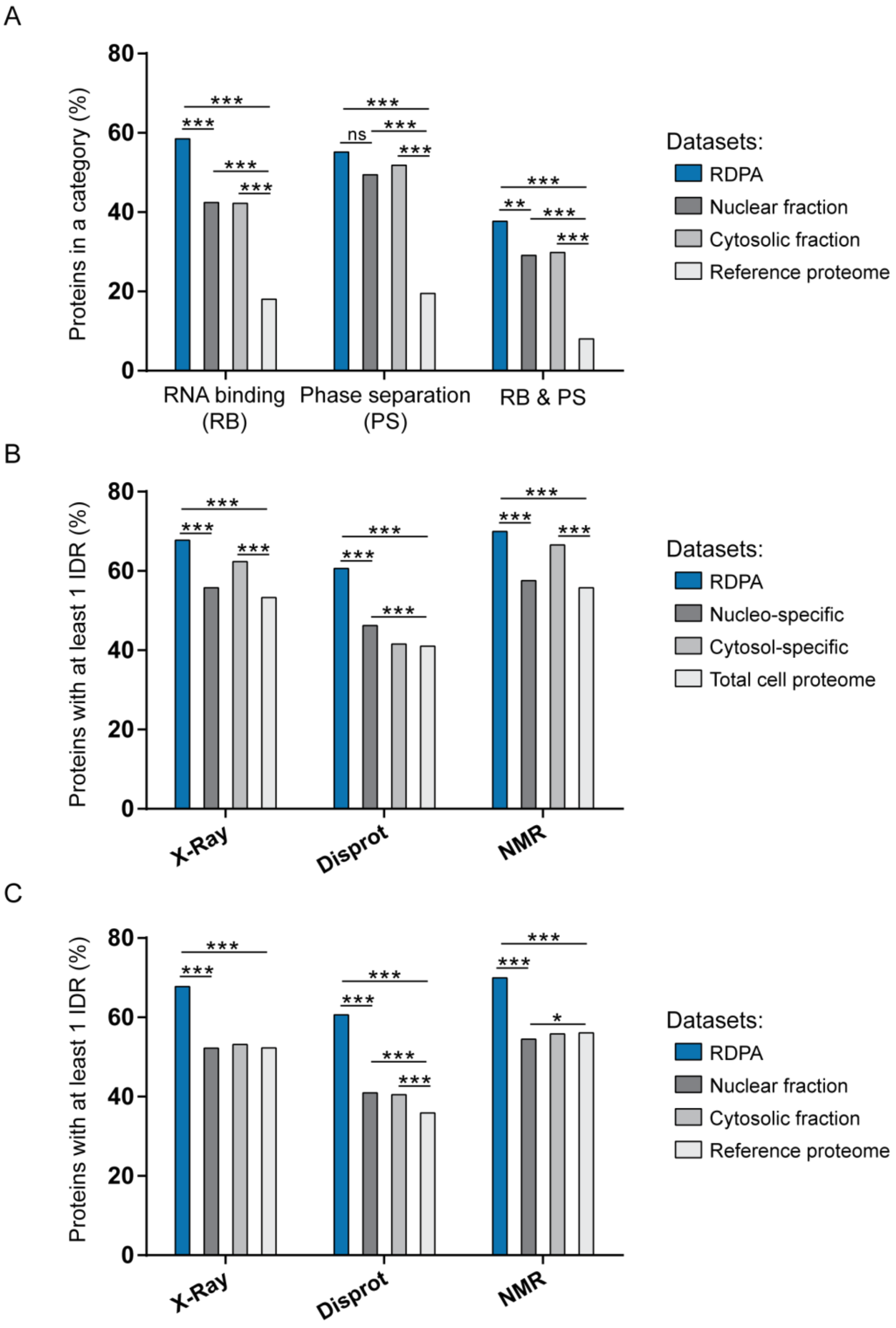

S4 Fig. Additional bioinformatic analyses of RDPA proteome features (related to Fig 2A and 2B). **A)** RDPA proteome is significantly enriched for RNA-binding, phase separation capacity, and combination of both properties. **B-C)** RDPA proteome is enriched for IDRs longer than 30 amino acid residues predicted by ESpritz X-Ray (X-Ray),

ESpritz Disprot (Disprot), and ESpritz NMR (NMR). Statistical analysis was performed using a hypergeometric test (ns not significant, * $P < 0.05$, ** $P < 0.01$, and *** $P < 0.001$).
