# Supplementary material for: The RNA-dependent association of phosphatidylinositol 4,5-bisphosphate with intrinsically disordered proteins contribute to nuclear compartmentalization
Source: PLoS Genet. 2024 Dec 2;20(12):e1011462. doi: 10.1371/journal.pgen.1011462 (PMC11668513; doi:10.1371/journal.pgen.1011462)
Supplement: S5 Fig — A-B) Enrichment of K/R motifs in the RDPA proteome. These motifs were abundantly present in the RDPA proteome, but only the K/R-x(3,7)-K-x-K/R-K/R motif (the longest one) was significantly enriched, compared to all other datasets. C) Percentage of PIP2-binding K/R motif sites localized in IDRs (from all K/R motif sites in the dataset) is elevated in RDPA proteome (only IDRs predicted by at least three different predictors with minimal length 20 amino acid residues were considered). Statistical analysis was performed using a hypergeometric test (* P < 0.05, ** P < 0.01, and *** P < 0.001). (PDF) [file pgen.1011462.s005.pdf]

S5 Fig

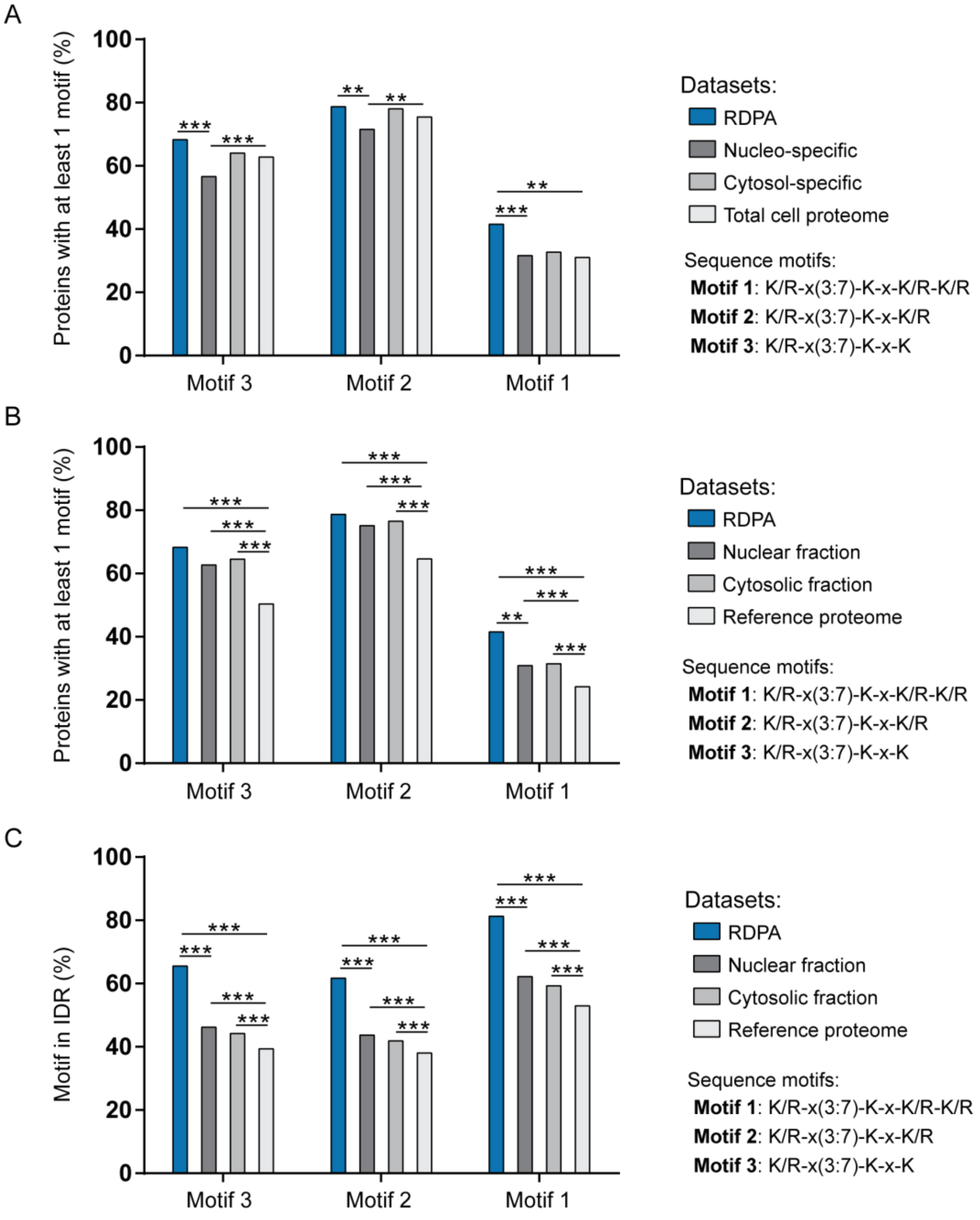

S5 Fig. Additional bioinformatic analyses of RDPA proteome features (related to Fig 2C). **A-B)** Enrichment of K/R motifs in the RDPA proteome. These motifs were abundantly present in the RDPA proteome, but only the K/R-x(3,7)-K-x-K/R-K/R motif (the longest one) was significantly enriched, compared to all other datasets. **C)** Percentage of PIP2-binding K/R motif sites localized in IDRs (from all K/R motif sites in the dataset) is elevated in RDPA proteome (only IDRs predicted by at least three different predictors with minimal length 20 amino acid residues were considered). Statistical analysis was performed using a hypergeometric test (* $P < 0.05$, ** $P < 0.01$, and *** $P < 0.001$).
