# Supplementary material for: The RNA-dependent association of phosphatidylinositol 4,5-bisphosphate with intrinsically disordered proteins contribute to nuclear compartmentalization
Source: PLoS Genet. 2024 Dec 2;20(12):e1011462. doi: 10.1371/journal.pgen.1011462 (PMC11668513; doi:10.1371/journal.pgen.1011462)
Supplement: S6 Fig — Bimodal pI distribution of IDRs predicted by nine different IDR predictors (Database of Disordered Protein Predictions - https://d2p2.pro/). Only IDRs with minimal length of 20 amino acid residues were considered. (PDF) [file pgen.1011462.s006.pdf]

S6 Fig

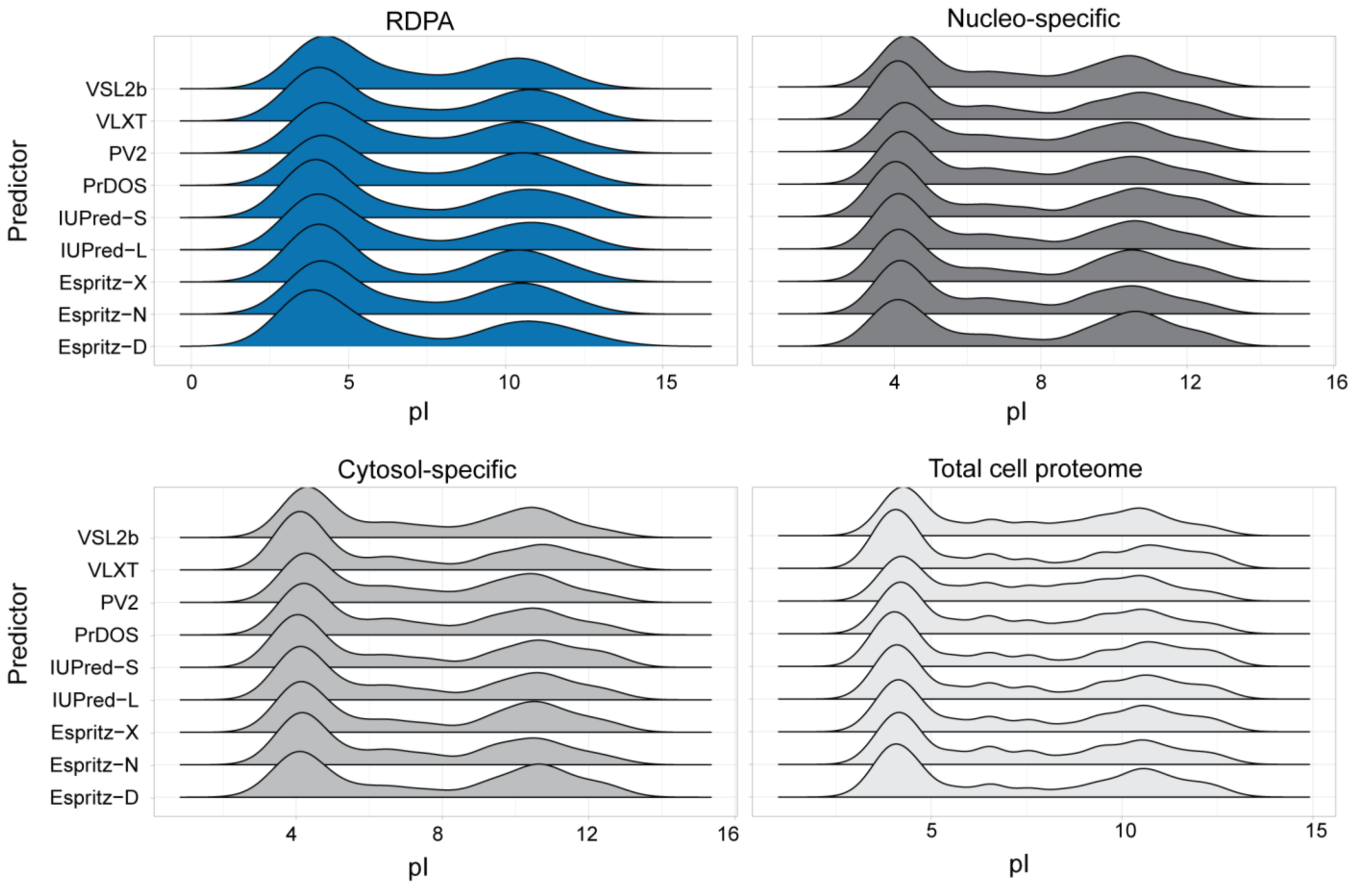

S6 Fig. Additional bioinformatic analysis of RDPA proteome features (relevant to Fig 2D). Bimodal pI distribution of IDRs predicted by nine different IDR predictors (Database of Disordered Protein Predictions - https://d2p2.pro/). Only IDRs with minimal length of 20 amino acid residues were considered.
