# Supplementary material for: The RNA-dependent association of phosphatidylinositol 4,5-bisphosphate with intrinsically disordered proteins contribute to nuclear compartmentalization
Source: PLoS Genet. 2024 Dec 2;20(12):e1011462. doi: 10.1371/journal.pgen.1011462 (PMC11668513; doi:10.1371/journal.pgen.1011462)
Supplement: S7 Fig — A) pI values of IDRs predicted by ESpritz X-Ray in the analyzed protein datasets show a bimodal distribution. B) The IDRs in the “acidic” population (pI < 7) are enriched with D/E amino acid residues. C) The IDRs in the “basic” population (pI > 7) are K/R-rich. (PDF) [file pgen.1011462.s007.pdf]

S7 Fig

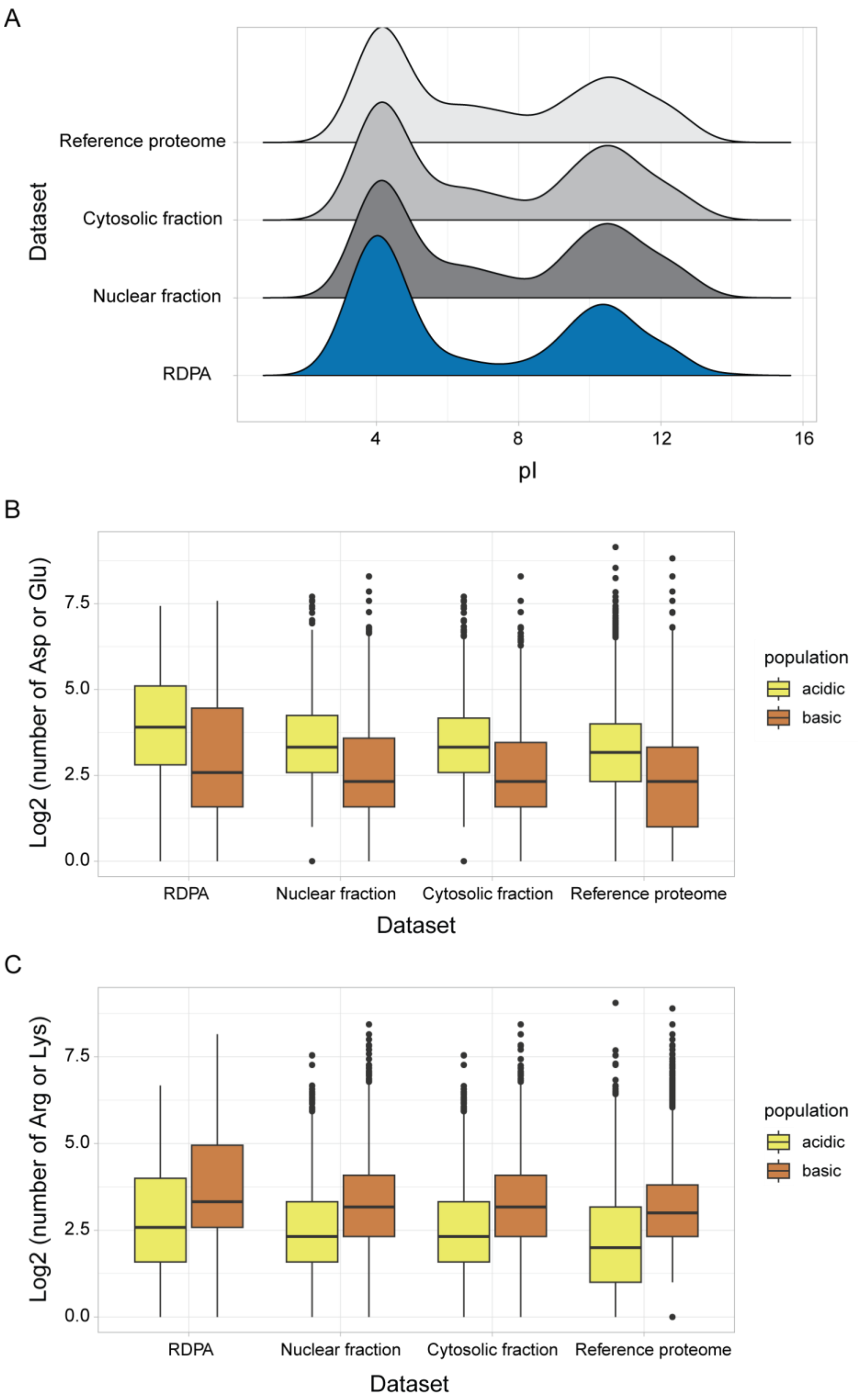

S7 Fig. Additional bioinformatic analyses of RDPA proteome features (relevant to Fig 2D-2F). A) pI values of IDRs predicted by ESpritz X-Ray in the analyzed protein datasets show a bimodal distribution. **B)** The IDRs in the "acidic" population (pI < 7) are enriched with D/E amino acid residues. **C)** The IDRs in the "basic" population (pI > 7) are K/R-rich**.**
