# Supplementary material for: The RNA-dependent association of phosphatidylinositol 4,5-bisphosphate with intrinsically disordered proteins contribute to nuclear compartmentalization
Source: PLoS Genet. 2024 Dec 2;20(12):e1011462. doi: 10.1371/journal.pgen.1011462 (PMC11668513; doi:10.1371/journal.pgen.1011462)
Supplement: S8 Fig — A-B) Distribution of the numbers of acidic (A) and basic (B) residues in IDRs predicted by nine different IDR predictors (Database of Disordered Protein Predictions; only IDRs with minimal length of 20 amino acid residues were considered) in the “main” datasets. C-D) Distribution of the numbers of acidic (C) and basic (D) residues in IDRs predicted by nine different IDR predictors (Database of Disordered Protein Predictions; only IDRs with minimal length of 20 amino acid residues were considered) in the “additional” datasets. (PDF) [file pgen.1011462.s008.pdf]

S8 Fig

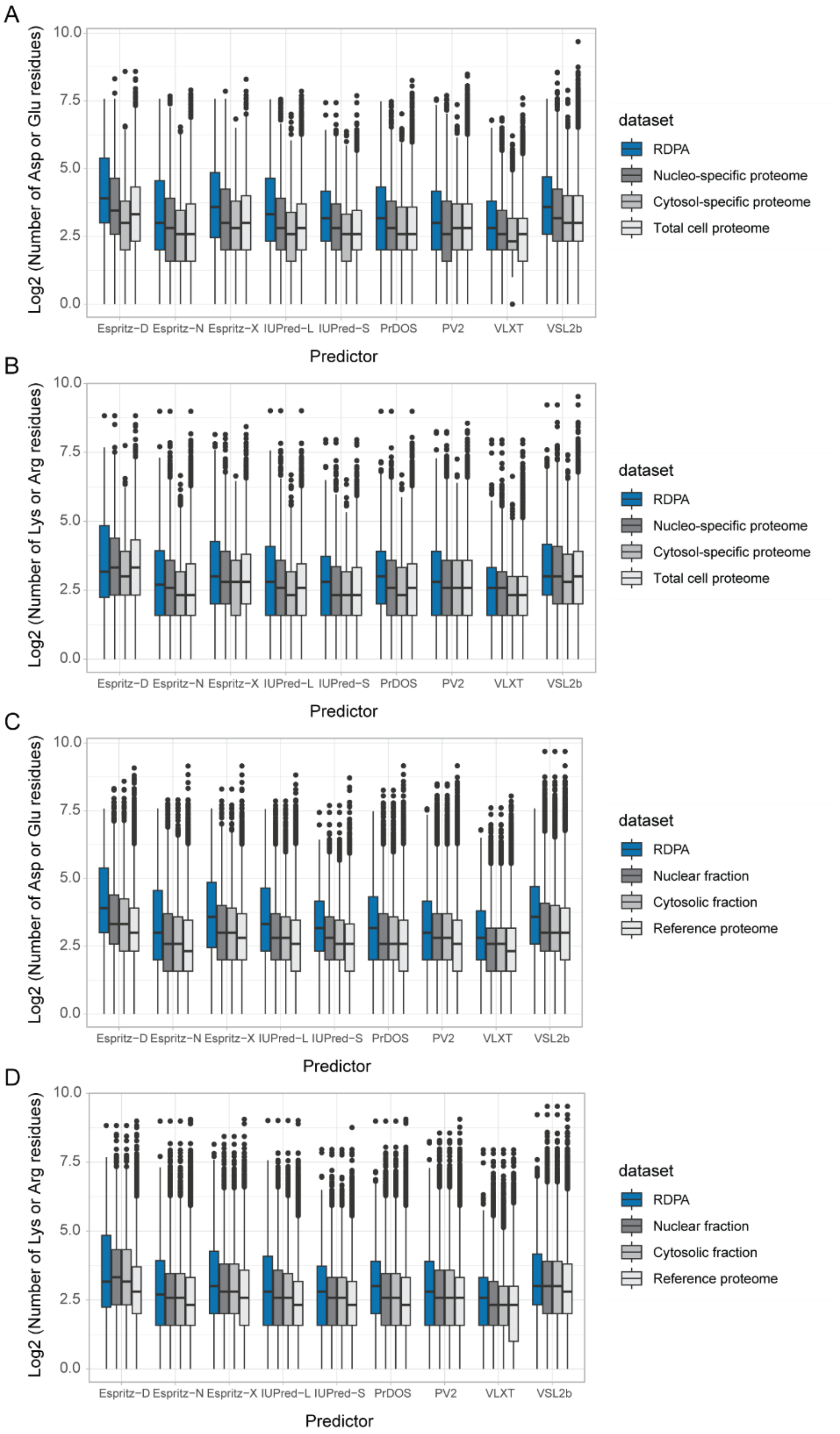

S8 Fig. Additional bioinformatic analyses of RDPA proteome features (relevant to Fig 2E and 2F). A-B) Distribution of the numbers of acidic **(A)** and basic **(B)** residues in IDRs predicted by nine different IDR predictors (Database of Disordered Protein Predictions; only IDRs with minimal length of 20 amino acid residues were considered) in the “main” datasets. **C-D)** Distribution of the numbers of acidic **(C)** and basic **(D)** residues in IDRs predicted by nine different IDR predictors (Database of Disordered Protein Predictions; only IDRs with minimal length of 20 amino acid residues were considered) in the “additional” datasets.
