# Supplementary material for: The RNA-dependent association of phosphatidylinositol 4,5-bisphosphate with intrinsically disordered proteins contribute to nuclear compartmentalization
Source: PLoS Genet. 2024 Dec 2;20(12):e1011462. doi: 10.1371/journal.pgen.1011462 (PMC11668513; doi:10.1371/journal.pgen.1011462)
Supplement: S9 Fig — A-C) Distribution of the log2 transformed length of all IDRs (A) or IDRs that were acidic (pI < 7) (B) or basic (pI > 7) (C) and predicted by nine different IDR predictors (Database of Disordered Protein Predictions; only IDRs with minimal length of 20 amino acid residues were considered) in the “main” datasets. (PDF) [file pgen.1011462.s009.pdf]

S9 Fig

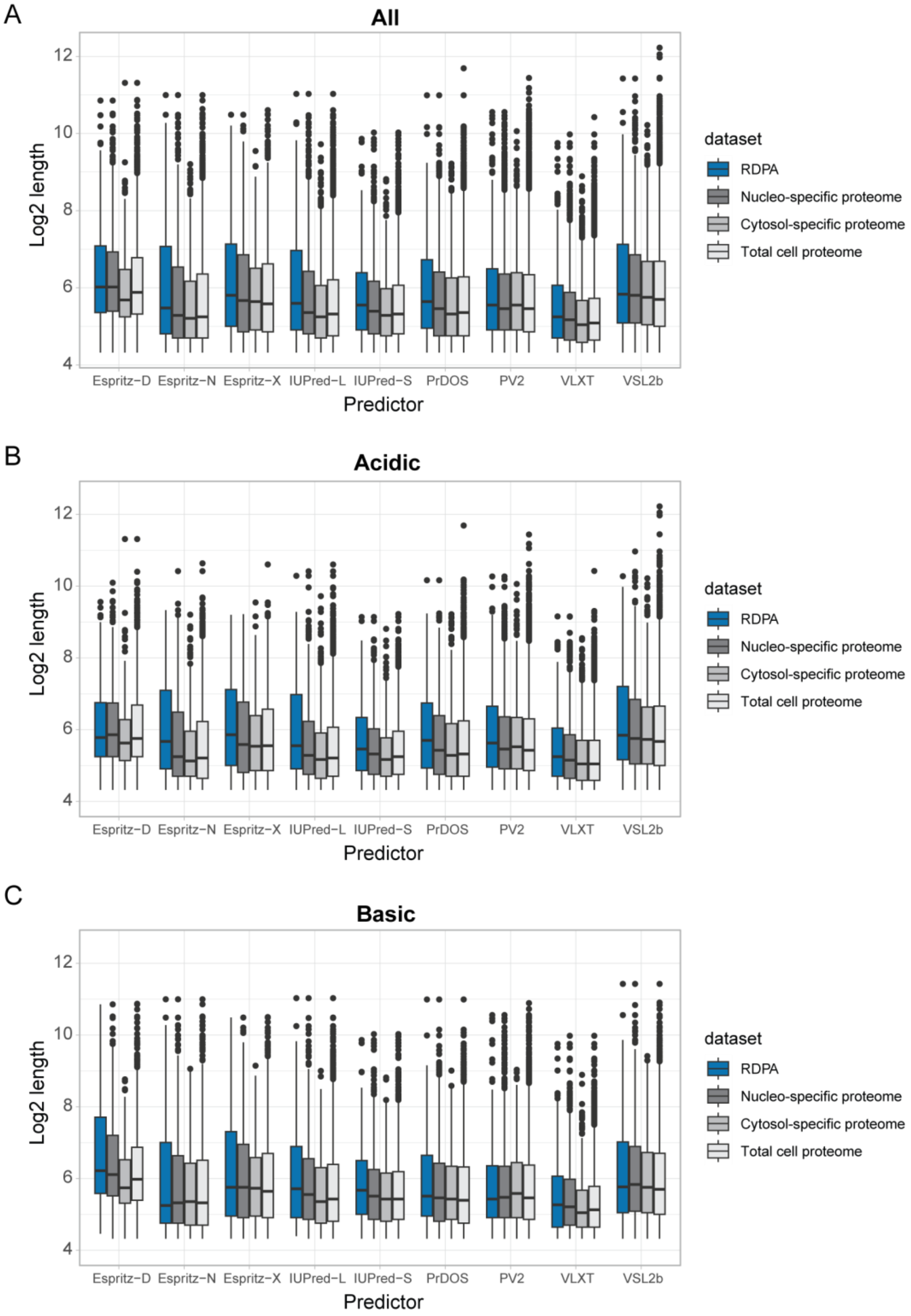

S9 Fig. Additional bioinformatic analysis of RDPA proteome features (relevant to Fig 2D and 2G). A-C) Distribution of the log2 transformed length of all IDRs **(A)** or IDRs that were acidic (pI < 7) **(B)** or basic (pI > 7) **(C)** and predicted by nine different IDR predictors (Database of Disordered Protein Predictions; only IDRs with minimal length of 20 amino acid residues were considered) in the “main” datasets.
