# Supplementary material for: The RNA-dependent association of phosphatidylinositol 4,5-bisphosphate with intrinsically disordered proteins contribute to nuclear compartmentalization
Source: PLoS Genet. 2024 Dec 2;20(12):e1011462. doi: 10.1371/journal.pgen.1011462 (PMC11668513; doi:10.1371/journal.pgen.1011462)
Supplement: S11 Fig — A-C) RDPA proteins IDRs containing the three K/R motifs tend to be significantly longer compared to the other six datasets. A) Boxplots show the distributions of log10 transformed length. B) Density plots of the IDR lengths highlight the presence of two populations of longer IDRs in the RDPA proteome and Nucleo-specific proteins (red arrows). C) The P values of all pairwise comparisons between the datasets and motifs were estimated by a pairwise Wilcox test. Benjamini-Hochberg correction was applied to correct for multiple hypothesis testing. Ref.–reference, prot.–proteome, fr.–fraction, spec.–specific. (PDF) [file pgen.1011462.s011.pdf]

S11 Fig

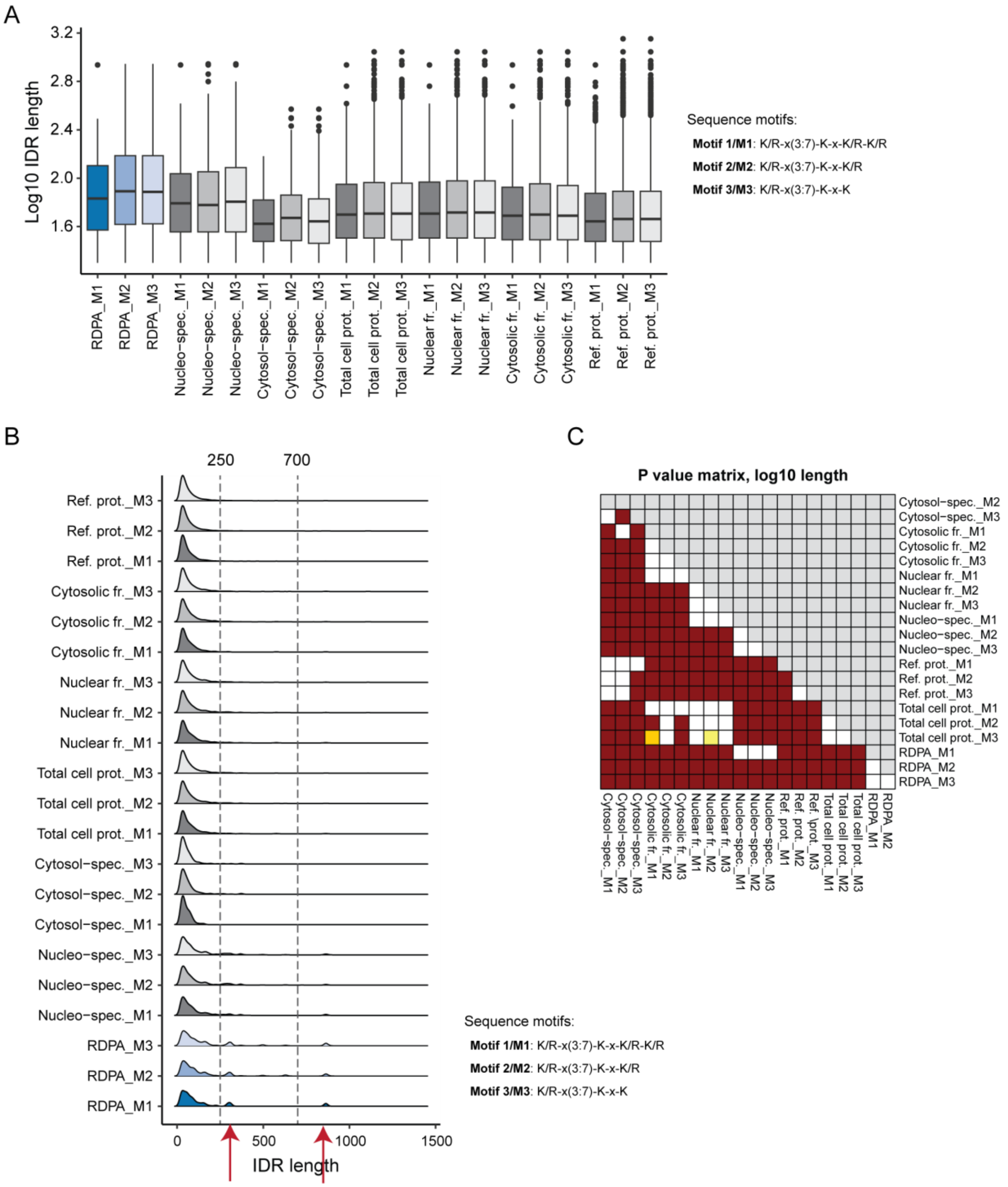

S11 Fig. Additional bioinformatic analysis of RDPA proteome features (relevant to Fig 2G). A-C) RDPA proteins IDRs containing the three K/R motifs tend to be significantly longer compared to the other six datasets. **A)** Boxplots show the distributions of log10 transformed length. **B)** Density plots of the IDR lengths highlight the presence of two populations of longer IDRs in the RDPA proteome and Nucleo-specific proteins (red arrows). **C)** The P values of all pairwise comparisons between the datasets and motifs were estimated by a pairwise Wilcox test. Benjamini-

Hochberg correction was applied to correct for multiple hypothesis testing. Ref. – reference, prot. – proteome, fr. – fraction, spec. – specific.
