# Supplementary material for: The RNA-dependent association of phosphatidylinositol 4,5-bisphosphate with intrinsically disordered proteins contribute to nuclear compartmentalization
Source: PLoS Genet. 2024 Dec 2;20(12):e1011462. doi: 10.1371/journal.pgen.1011462 (PMC11668513; doi:10.1371/journal.pgen.1011462)
Supplement: S12 Fig — The percentage of disordered amino acid residues in IDRs predicted by ESpritz X-Ray (X-Ray), ESpritz Disprot (Disprot), and ESpritz NMR (NMR) in the “main” (A) and “additional” (B) datasets. Statistical analysis was performed using a hypergeometric test (*** P < 0.001). (PDF) [file pgen.1011462.s012.pdf]

S12 Fig

A

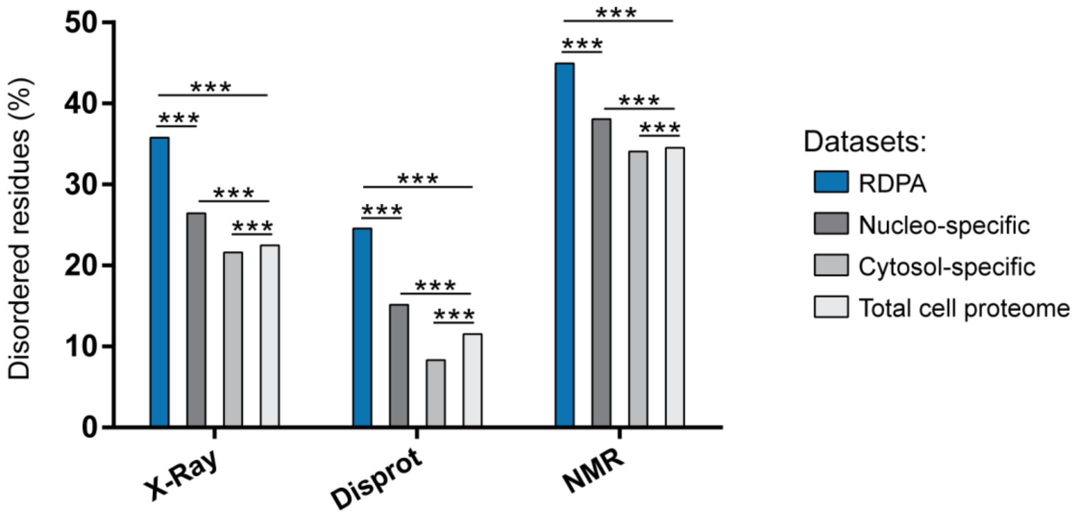

B

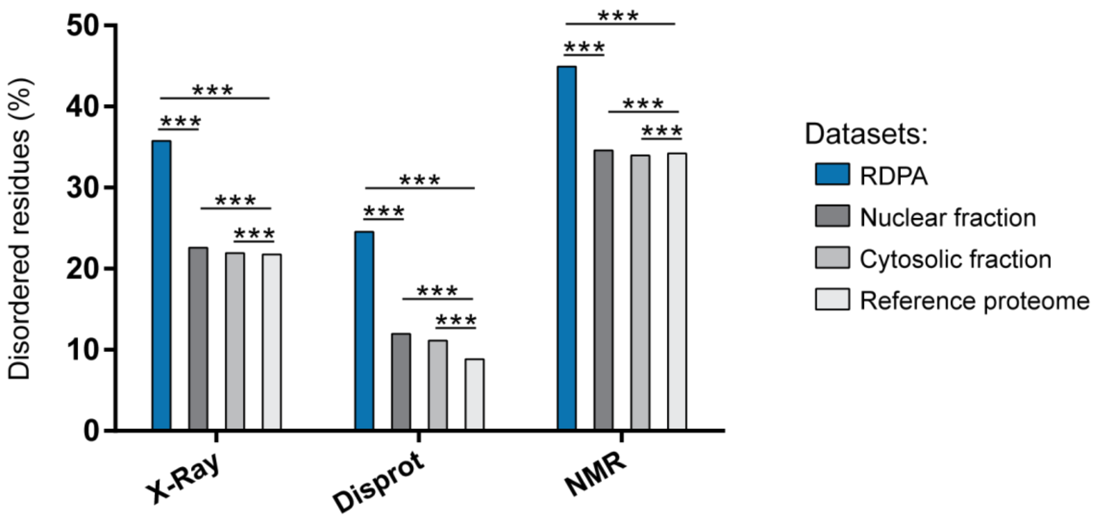

S12 Fig. Additional bioinformatic analysis of RDPA proteome features (relevant to Fig 2B and 2G). The percentage of disordered amino acid residues in IDRs predicted by ESpritz X-Ray (X-Ray), ESpritz Disprot (Disprot), and ESpritz NMR (NMR) in the "main" **(A)** and "additional" **(B)** datasets. Statistical analysis was performed using a hypergeometric test (*** $P < 0.001$).
