# Supplementary material for: The RNA-dependent association of phosphatidylinositol 4,5-bisphosphate with intrinsically disordered proteins contribute to nuclear compartmentalization
Source: PLoS Genet. 2024 Dec 2;20(12):e1011462. doi: 10.1371/journal.pgen.1011462 (PMC11668513; doi:10.1371/journal.pgen.1011462)
Supplement: S13 Fig — A) Boxplots show the distributions of pI values of IDRs between datasets and K/R motifs. B) Density plots of the IDR pI values highlight the presence of bimodal distributions. C) The P values of all pairwise comparisons between the datasets and motifs were estimated by a pairwise Wilcox test. Benjamini-Hochberg correction was applied to correct for multiple hypothesis testing. Ref.–reference, prot.–proteome, fr.–fraction, spec.–specific. (PDF) [file pgen.1011462.s013.pdf]

S13 Fig

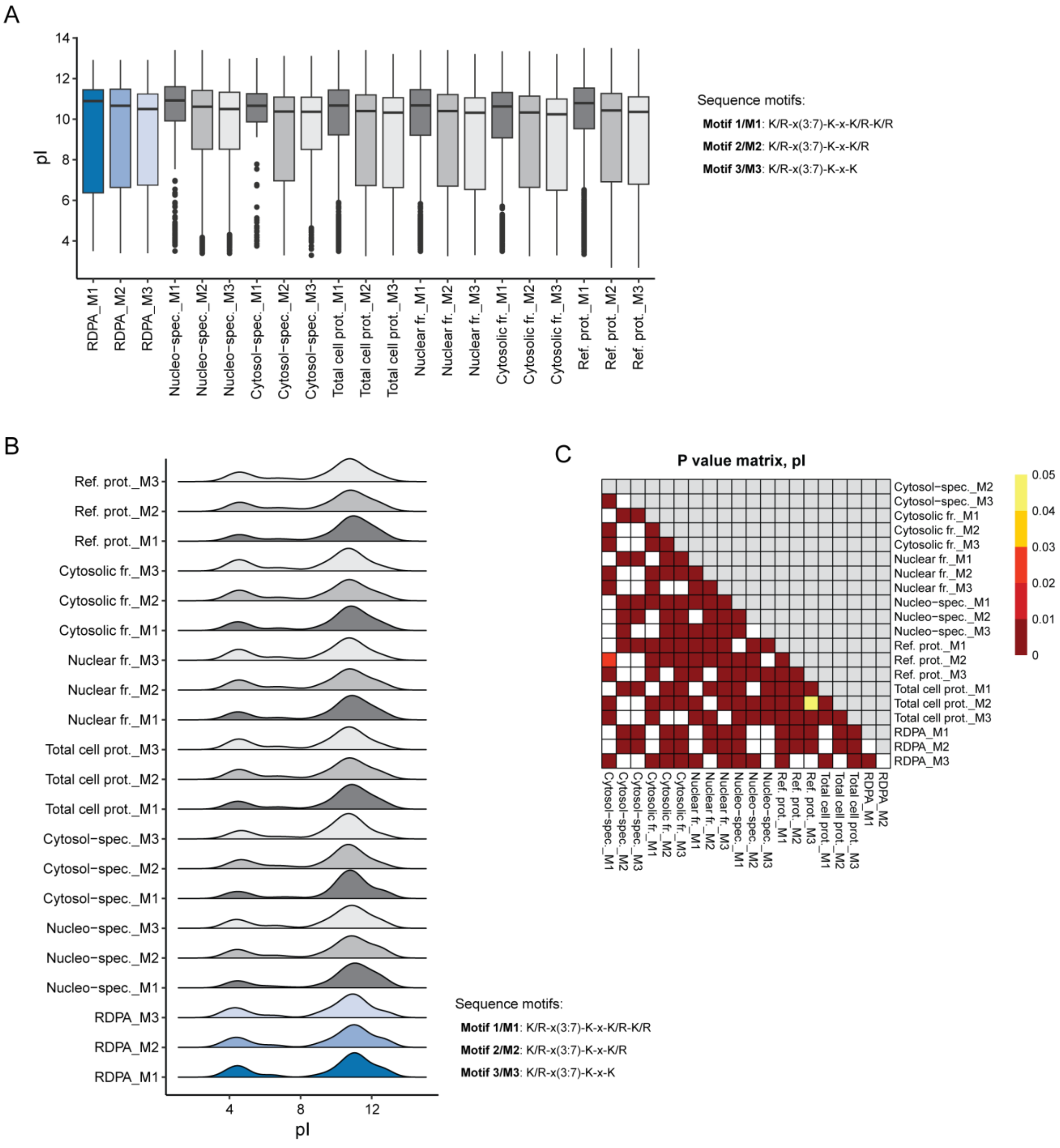

S13 Fig. Additional bioinformatic analysis of RDPA proteome features (relevant to Fig 2H). A) Boxplots show the distributions of pI values of IDRs between datasets and K/R motifs. **B)** Density plots of the IDR pI values highlight the presence of bimodal distributions. **C)** The P values of all pairwise comparisons between the datasets and motifs were estimated by a pairwise Wilcox test. Benjamini-Hochberg correction was applied to correct for multiple hypothesis testing. Ref. – reference, prot. – proteome, fr. – fraction, spec. – specific.
