# Supplementary material for: The RNA-dependent association of phosphatidylinositol 4,5-bisphosphate with intrinsically disordered proteins contribute to nuclear compartmentalization
Source: PLoS Genet. 2024 Dec 2;20(12):e1011462. doi: 10.1371/journal.pgen.1011462 (PMC11668513; doi:10.1371/journal.pgen.1011462)
Supplement: S14 Fig — A-B) Distribution of the GRAVY scores of IDRs predicted by nine different IDR predictors (Database of Disordered Protein Predictions; only IDRs with minimal length of 20 amino acid residues were considered) in the “main” (A) and “additional” (B) datasets. (PDF) [file pgen.1011462.s014.pdf]

S14 Fig

A

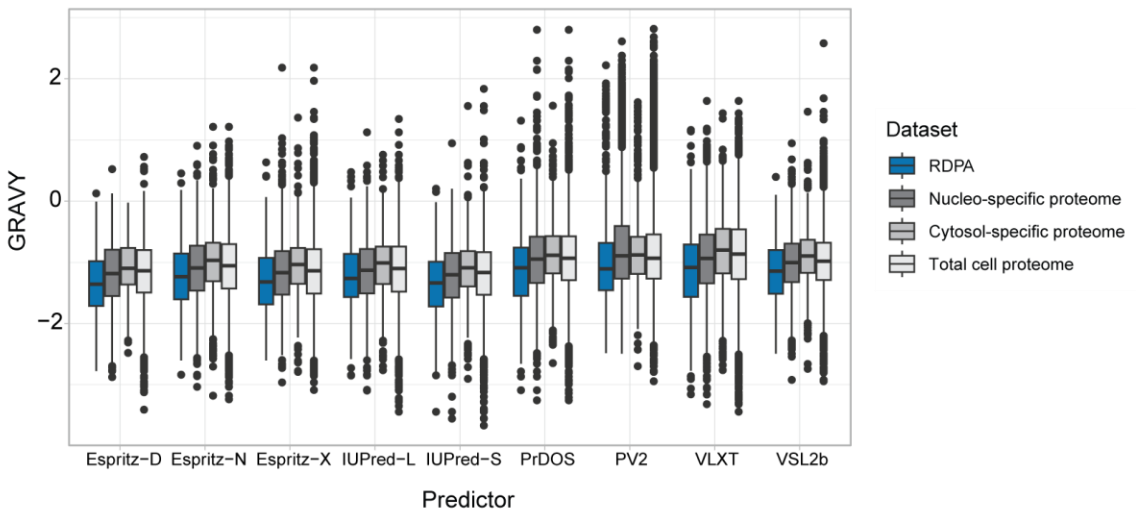

B

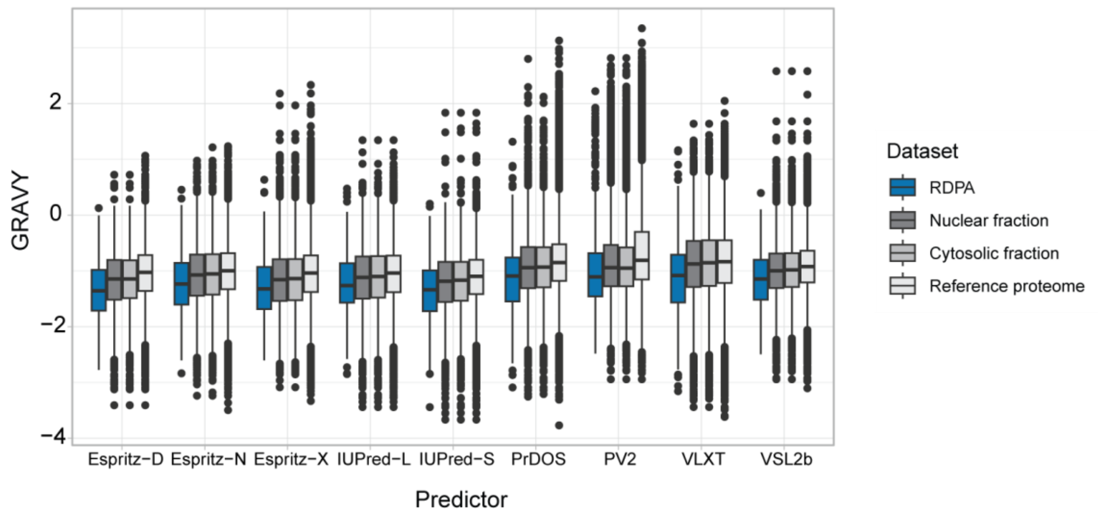

S14 Fig. Additional bioinformatic analysis of RDPA proteome features (relevant to Fig 2I). A-B) Distribution of the GRAVY scores of IDRs predicted by nine different IDR predictors (Database of Disordered Protein Predictions; only IDRs with minimal length of 20 amino acid residues were considered) in the “main” **(A)** and “additional” **(B)** datasets.
