# Supplementary material for: The RNA-dependent association of phosphatidylinositol 4,5-bisphosphate with intrinsically disordered proteins contribute to nuclear compartmentalization
Source: PLoS Genet. 2024 Dec 2;20(12):e1011462. doi: 10.1371/journal.pgen.1011462 (PMC11668513; doi:10.1371/journal.pgen.1011462)
Supplement: S17 Fig — A-C) Gene ontology (GO) analysis of human proteins containing K/R-x(3,7)-K-x-K motif in IDRs using SLiMSearch tool based on (A) cellular compartment (GOCC), (B) molecular function (GOMF), and (C) biological process (GOBP). The y-axis shows the -log10 adjusted p-value (Fisher’s exact test) of proteins from a GO category, the x-axis shows the log2 enrichment factor. The size of the bubble corresponds to the number of proteins. (PDF) [file pgen.1011462.s017.pdf]

S17 Fig

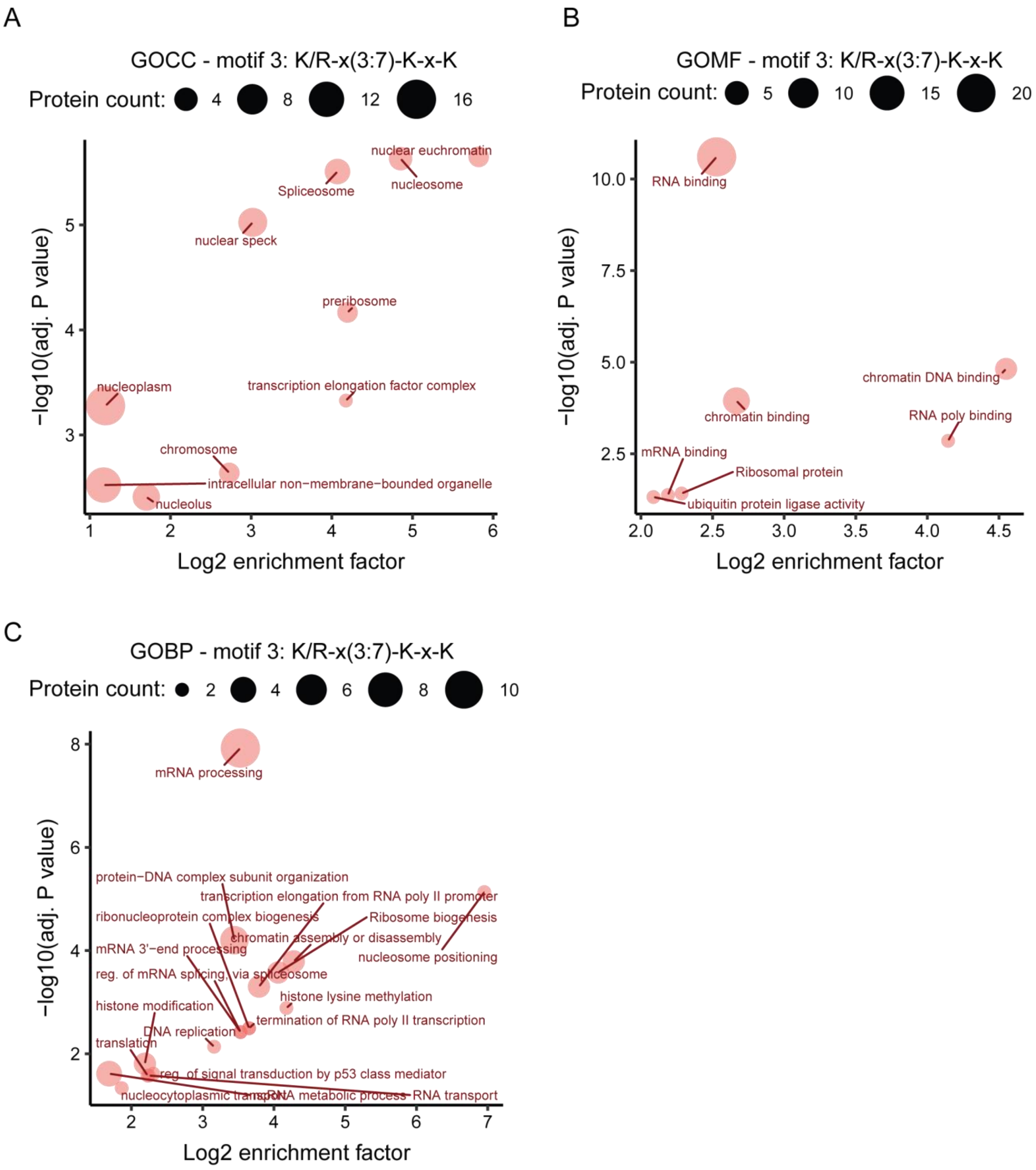

S17 Fig. Functional analysis of the RDPA proteome (relevant to Fig 4B-4D). A-C) Gene ontology (GO) analysis of human proteins containing K/R-x(3,7)-K-x-K motif in IDRs using SLiMSearch tool based on **(A)** cellular compartment (GOCC), **(B)** molecular function (GOMF), and **(C)** biological process (GOBP). The y-axis shows the -log10 adjusted

p-value (Fisher's exact test) of proteins from a GO category, the x-axis shows the log2 enrichment factor. The size of the bubble corresponds to the number of proteins.
