# Supplementary material for: The RNA-dependent association of phosphatidylinositol 4,5-bisphosphate with intrinsically disordered proteins contribute to nuclear compartmentalization
Source: PLoS Genet. 2024 Dec 2;20(12):e1011462. doi: 10.1371/journal.pgen.1011462 (PMC11668513; doi:10.1371/journal.pgen.1011462)
Supplement: S18 Fig — The PIP2 and empty control beads were incubated for 1 h at 4°C in nuclear lysates, washed, and subjected to WB detection of CAND1 protein. WB signals at each pull-down condition in every repetition were normalized to the signal at PIP2 pull-down upon RNA addition condition. Statistical analysis was performed using Student’s t-test (n = 3). Error bars correspond to SEM (NL–nuclear lysate, * P < 0.05, ** P < 0.001, *** P < 0.0005, **** P < 0.0001). The following treatments were used in the respective specimens as indicated in Fig 4A and 4B: the addition of 30 μg of nuclear RNA extract, 300 mM NaCl, 100 mM NH4OAc, 10% 1,6-hexanediol, and 10% dextran. (PDF) [file pgen.1011462.s018.pdf]

S18 Fig

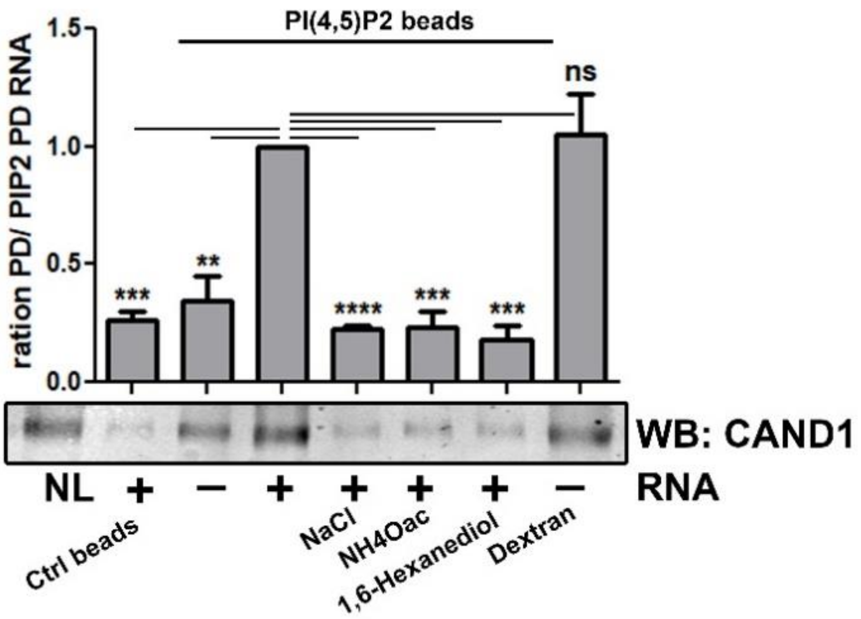

S18 Fig. PIP2-conjugated agarose beads pull-down assays from nuclear lysates with added nuclear RNA extract upon different conditions. The PIP2 and empty control beads were incubated for 1 h at 4 °C in nuclear lysates, washed, and subjected to WB detection of CAND1 protein. WB signals at each pull-down condition in every repetition were normalized to the signal at PIP2 pull-down upon RNA addition condition. Statistical analysis was performed using Student's t-test (n = 3). Error bars correspond to SEM (NL – nuclear lysate, * $P < 0.05$, ** $P < 0.001$, *** $P < 0.0005$, **** $P < 0.0001$). The following treatments were used in the respective specimens as indicated in Fig 4A and 4B: the addition of 30 µg of nuclear RNA extract, 300 mM NaCl, 100 mM NH_4OAc, 10% 1,6-hexanediol, and 10% dextran.
