# Supplementary material for: The RNA-dependent association of phosphatidylinositol 4,5-bisphosphate with intrinsically disordered proteins contribute to nuclear compartmentalization
Source: PLoS Genet. 2024 Dec 2;20(12):e1011462. doi: 10.1371/journal.pgen.1011462 (PMC11668513; doi:10.1371/journal.pgen.1011462)
Supplement: S19 Fig — PIP2 beads were incubated in nuclear lysates for 1 h at 4°C, washed, and subjected to WB detection of BRD4 protein. WB signals for each pull-down condition in each replicate were normalized to the highest signal (25 mM Mg2+). Statistical analysis was performed by Student’s t-test (n = 3). Error bars correspond to SEM. (PDF) [file pgen.1011462.s019.pdf]

S19 Fig

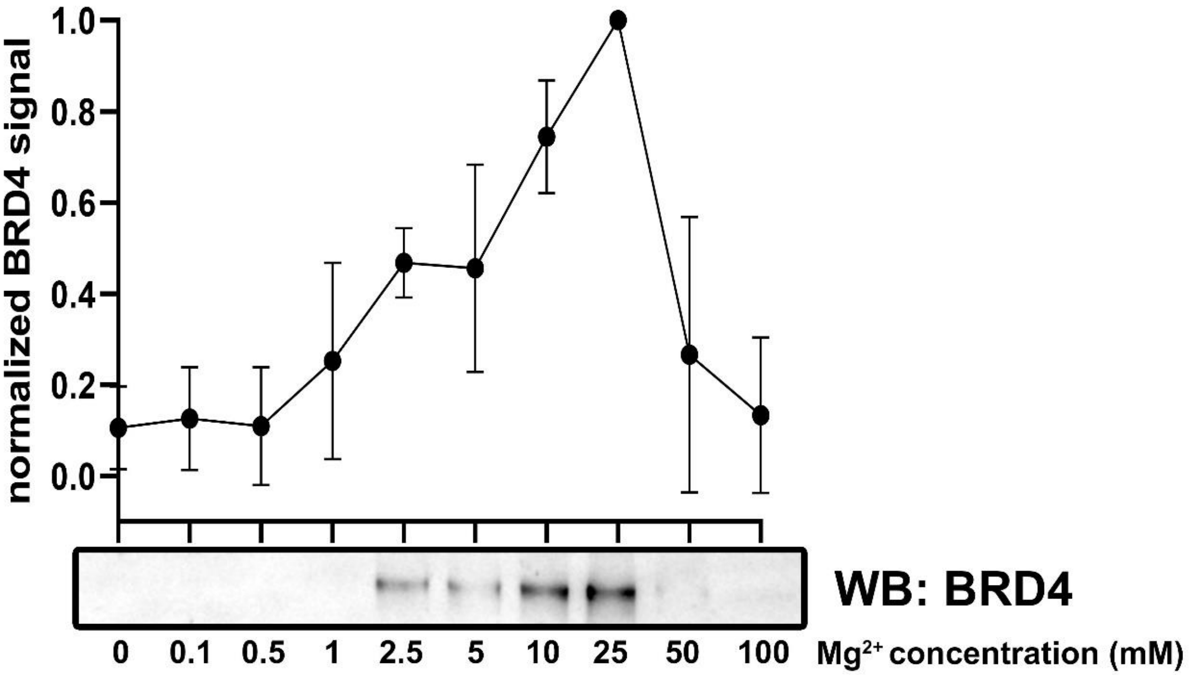

S19 Fig. PIP2-conjugated agarose beads pull-down assays from nuclear lysates with the addition of 30 µg nuclear RNA extract at increasing concentrations of Mg^{2+}. PIP2 beads were incubated in nuclear lysates for 1 h at 4 °C, washed, and subjected to WB detection of BRD4 protein. WB signals for each pull-down condition in each replicate were normalized to the highest signal (25 mM Mg^{2+}). Statistical analysis was performed by Student's t-test (n = 3). Error bars correspond to SEM.
