# Supplementary material for: The RNA-dependent association of phosphatidylinositol 4,5-bisphosphate with intrinsically disordered proteins contribute to nuclear compartmentalization
Source: PLoS Genet. 2024 Dec 2;20(12):e1011462. doi: 10.1371/journal.pgen.1011462 (PMC11668513; doi:10.1371/journal.pgen.1011462)
Supplement: S20 Fig — PIPs and control empty beads were incubated in nuclear lysates for 1 h at 4°C, washed, and subjected to WB detection of BRD4 protein. WB signals for each pull-down condition in each replicate were normalized to the highest signal (PI(4,5)P2). Statistical analysis was performed by Student’s t-test (n = 4). Error bars correspond to SEM (** P < 0.001, **** P < 0.0001). (PDF) [file pgen.1011462.s020.pdf]

S20 Fig

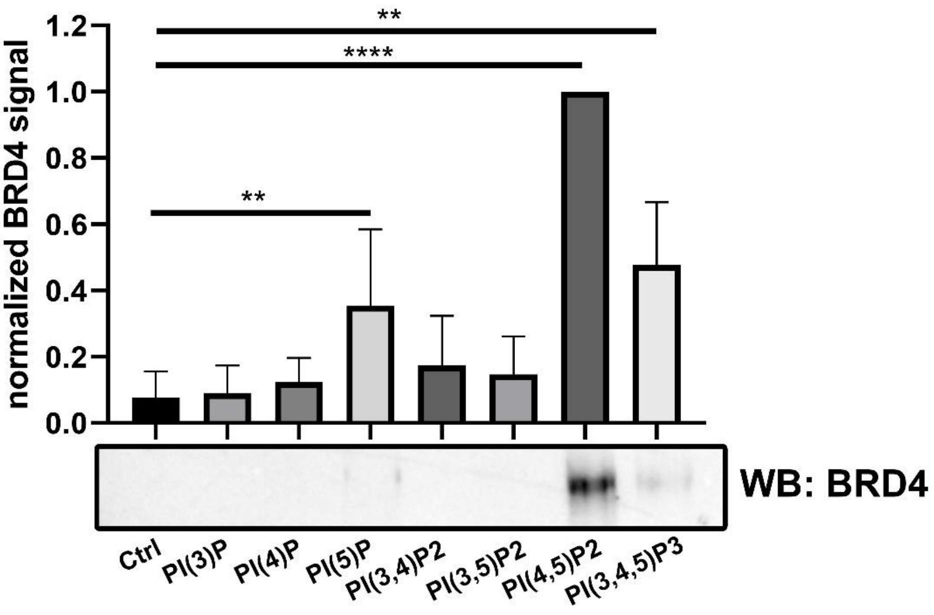

S20 Fig. Different PIPs-conjugated agarose beads pull-down assays from nuclear lysates with the addition of 30 µg nuclear RNA extract. PIPs and control empty beads were incubated in nuclear lysates for 1 h at 4 °C, washed, and subjected to WB detection of BRD4 protein. WB signals for each pull-down condition in each replicate were normalized to the highest signal (PI(4,5)P2). Statistical analysis was performed by Student's t-test ($n = 4$). Error bars correspond to SEM (** $P < 0.001$, **** $P < 0.0001$).
