# Supplementary material for: The RNA-dependent association of phosphatidylinositol 4,5-bisphosphate with intrinsically disordered proteins contribute to nuclear compartmentalization
Source: PLoS Genet. 2024 Dec 2;20(12):e1011462. doi: 10.1371/journal.pgen.1011462 (PMC11668513; doi:10.1371/journal.pgen.1011462)
Supplement: S21 Fig — Manipulation of PIP2 level by PIP5KA and SHIP2 knock-down (relevant to Fig 5C and 5D). (A) Microscopy confirmation of the manipulation of PIP2 levels induced by depletion of PIP5KA and SHIP2 enzymes. Statistical analysis was performed using Student’s t-tests (**** P < 0.0001), n = 3, n = 4, N = 37 KD control cells, KD PIP5KA N = 44, and KD SHIP2 N = 50 cells, respectively). Error bars correspond to SEM. (B) WB analysis of the efficacy of PIP5KA and SHIP2 siRNA depletion and its effect on BRD4 protein levels. (PDF) [file pgen.1011462.s021.pdf]

S21 Fig

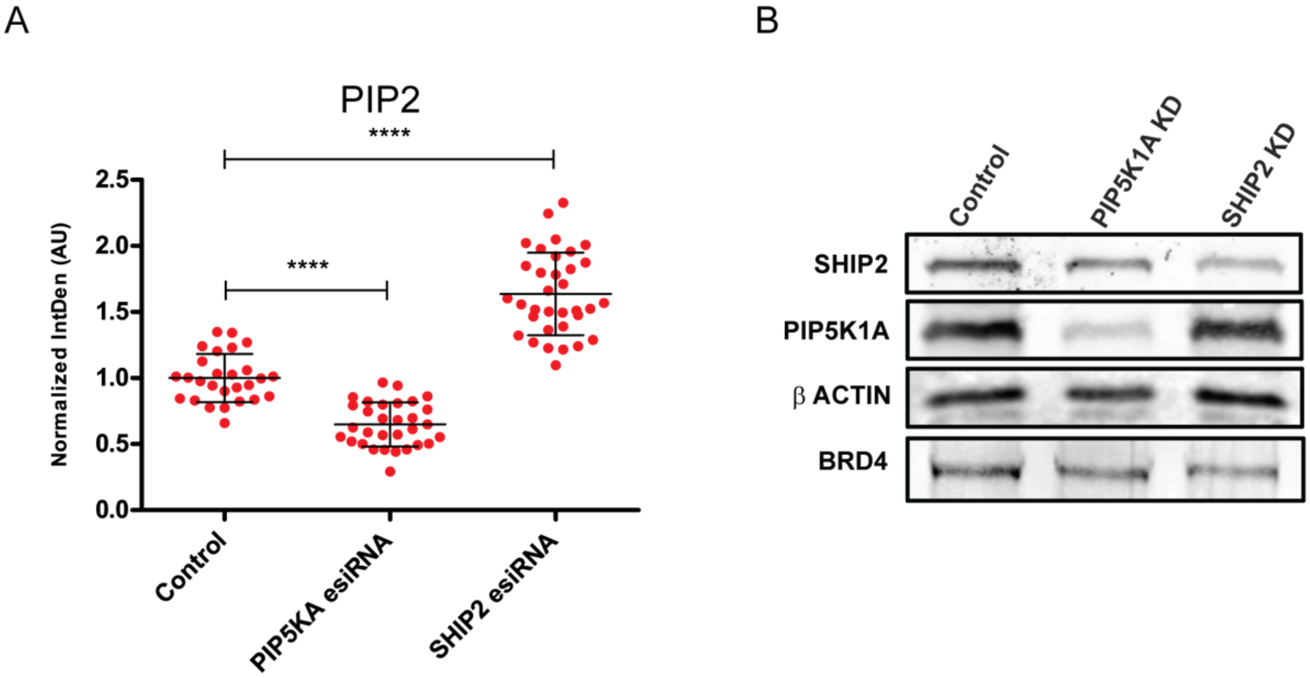

S21 Fig. Manipulation of PIP2 level by PIP5KA and SHIP2 knock-down (relevant to Fig 5C and 5D). (A) Microscopy confirmation of the manipulation of PIP2 levels induced by depletion of PIP5KA and SHIP2 enzymes. Statistical analysis was performed using Student's t-tests (**** $P < 0.0001$), n = 3, n = 4, N = 37 KD control cells, KD PIP5KA N = 44, and KD SHIP2 N = 50 cells, respectively). Error bars correspond to SEM. **(B)** WB analysis of the efficacy of PIP5KA and SHIP2 siRNA depletion and its effect on BRD4 protein levels.
