# Supplementary material for: The RNA-dependent association of phosphatidylinositol 4,5-bisphosphate with intrinsically disordered proteins contribute to nuclear compartmentalization
Source: PLoS Genet. 2024 Dec 2;20(12):e1011462. doi: 10.1371/journal.pgen.1011462 (PMC11668513; doi:10.1371/journal.pgen.1011462)
Supplement: S22 Fig — (A) Representative figures show the localization of PIP2 and BRD4 using immunofluorescence staining. The last column shows the identified foci in false red color, which does not represent the intensity of the signal. Scale bars correspond to 5 μm. B) Quantification of PIP2 levels upon K149 treatment in U2OS cells. (C) The chart visualizes the average number of BRD4 foci identified per cell in control and SHIP2 inhibited U2OS cells. Statistical analysis was performed using Student’s t-tests (**** P < 0.0001), n = 5, N = 56 control cells, N = 62 SHIP2 inhibited cells). Error bars correspond to SEM. (PDF) [file pgen.1011462.s022.pdf]

S22 Fig

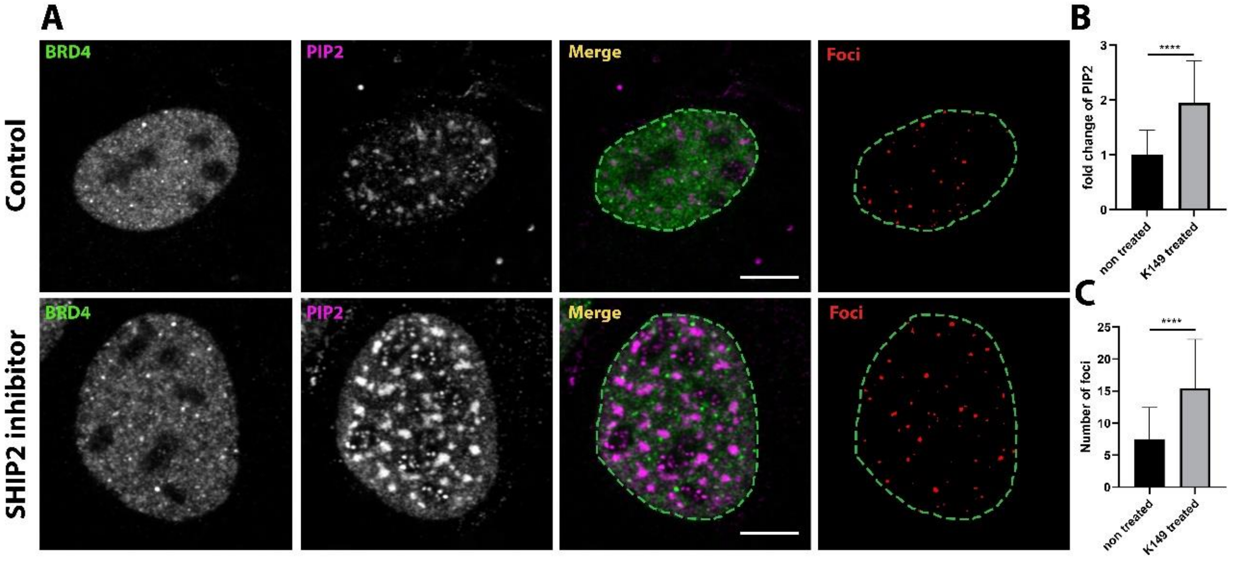

S22 Fig. Confocal microscopy visualization of changes in the number of BRD4 foci induced by SHIP2 inhibition. (A) Representative figures show the localization of PIP2 and BRD4 using immunofluorescence staining. The last column shows the identified foci in false red color, which does not represent the intensity of the signal. Scale bars correspond to 5 µm. **B)** Quantification of PIP2 levels upon K149 treatment in U2OS cells. **(C)** The chart visualizes the average number of BRD4 foci identified per cell in control and SHIP2 inhibited U2OS cells. Statistical analysis was performed using Student's t-tests (**** $P < 0.0001$), n = 5, N = 56 control cells, N = 62 SHIP2 inhibited cells). Error bars correspond to SEM.
