# Supplementary material for: The RNA-dependent association of phosphatidylinositol 4,5-bisphosphate with intrinsically disordered proteins contribute to nuclear compartmentalization
Source: PLoS Genet. 2024 Dec 2;20(12):e1011462. doi: 10.1371/journal.pgen.1011462 (PMC11668513; doi:10.1371/journal.pgen.1011462)
Supplement: S23 Fig — (A) Representative figures show the localization of PIP2 and BRD4 using immunofluorescence staining. The last column shows the identified foci in false red color, which does not represent the intensity of the signal. Scale bars correspond to 5 μm. B) The chart visualizes the average number of BRD4 foci identified per cell in non-treated and RNase III treated semi-permeabilized U2OS cells. Statistical analysis was performed using Student’s t-tests (**** P < 0.0001), n = 4, N = 46 non-treated cells, N = 64 RNase III treated cells). Error bars correspond to SEM. (PDF) [file pgen.1011462.s023.pdf]

S23 Fig

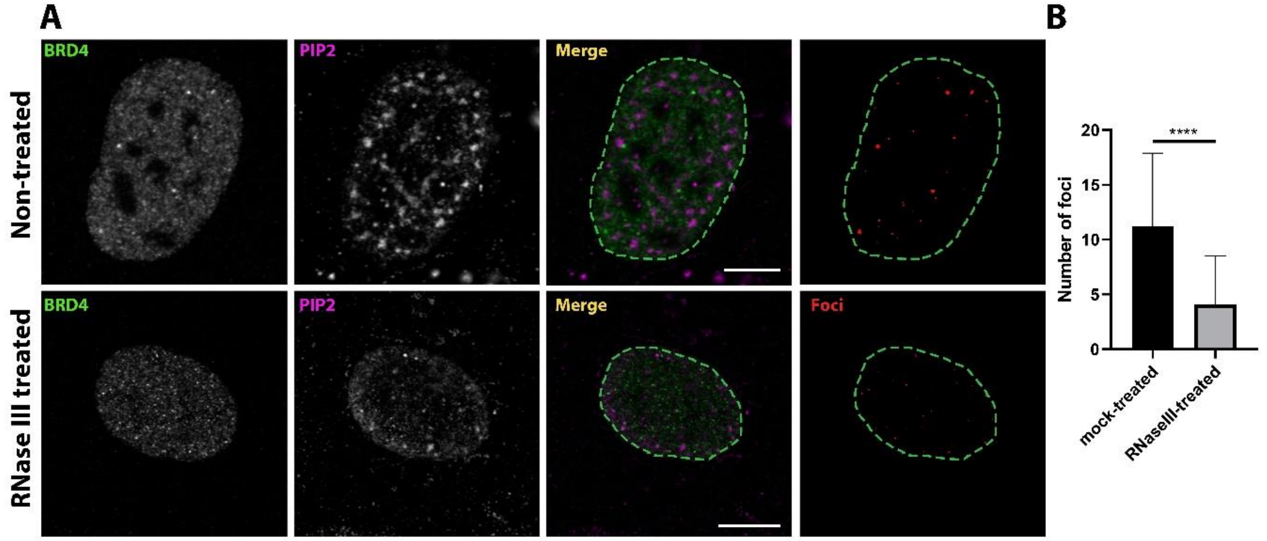

S23 Fig. Changes in the number of BRD4 foci induced by the RNase III treatment in semi-permeabilized U2OS cells visualized by confocal microscopy. (A) Representative figures show the localization of PIP2 and BRD4 using immunofluorescence staining. The last column shows the identified foci in false red color, which does not represent the intensity of the signal. Scale bars correspond to 5 µm. **B)** The chart visualizes the average number of BRD4 foci identified per cell in non-treated and RNase III treated semi-permeabilized U2OS cells. Statistical analysis was performed using Student's t-tests (**** $P < 0.0001$), n = 4, N = 46 non-treated cells, N = 64 RNase III treated cells). Error bars correspond to SEM.
